# Deletion of the Huntingtin Polyglutamine Stretch Enhances Neuronal Autophagy and Longevity in Mice

**DOI:** 10.1371/journal.pgen.1000838

**Published:** 2010-02-05

**Authors:** Shuqiu Zheng, Erin B. D. Clabough, Sovan Sarkar, Marie Futter, David C. Rubinsztein, Scott O. Zeitlin

**Affiliations:** 1 Department of Neuroscience, University of Virginia School of Medicine, Charlottesville, Virginia, United States of America; 2 Department of Medical Genetics, University of Cambridge, Cambridge Institute for Medical Research, Addenbrooke's Hospital, Cambridge, United Kingdom; Massachusetts General Hospital, United States of America

## Abstract

Expansion of a stretch of polyglutamine in huntingtin (htt), the protein product of the IT15 gene, causes Huntington's disease (HD). Previous investigations into the role of the polyglutamine stretch (polyQ) in htt function have suggested that its length may modulate a normal htt function involved in regulating energy homeostasis. Here we show that expression of full-length htt lacking its polyglutamine stretch (ΔQ-htt) in a knockin mouse model for HD (*Hdh^140Q/ΔQ^*), reduces significantly neuropil mutant htt aggregates, ameliorates motor/behavioral deficits, and extends lifespan in comparison to the HD model mice (*Hdh^140Q/+^*). The rescue of HD model phenotypes is accompanied by the normalization of lipofuscin levels in the brain and an increase in the steady-state levels of the mammalian autophagy marker microtubule-associate protein 1 light chain 3-II (LC3-II). We also find that ΔQ-htt expression in vitro increases autophagosome synthesis and stimulates the Atg5-dependent clearance of truncated N-terminal htt aggregates. ΔQ-htt's effect on autophagy most likely represents a gain-of-function, as overexpression of full-length wild-type htt in vitro does not increase autophagosome synthesis. Moreover, *Hdh^ΔQ/ΔQ^* mice live significantly longer than wild-type mice, suggesting that autophagy upregulation may be beneficial both in diseases caused by toxic intracellular aggregate-prone proteins and also as a lifespan extender in normal mammals.

## Author Summary

Expansion of a stretch of glutamines near the amino-terminus of huntingtin (htt), the protein product of the IT15 gene, is a deleterious mutation that causes Huntington's disease (HD). Here we show, in contrast, that deletion of htt's normal polyglutamine stretch (ΔQ-htt) is a potentially beneficial mutation that can ameliorate HD mouse model phenotypes when ΔQ-htt is expressed together with a version of htt with the HD mutation. In addition, ΔQ-htt expression can enhance longevity when expressed in either an HD mouse model or in non–HD mice. ΔQ-htt's effects on both lifespan and HD model phenotypes are likely due to an increase in autophagy, a major recycling pathway in cells that is involved in the turnover of cellular components, and aggregated protein. Based on our results, we suggest that development of therapeutic agents that can stimulate autophagy may help both in treating neurodegenerative disorders like HD and also in increasing longevity.

## Introduction

In vertebrates, the polyQ stretch within htt is located close to the protein's N-terminus, and separates a highly conserved 17 amino acid N-terminal domain (N1–17) that can act as a membrane association signal [1], from a proline-rich region that is implicated in protein-protein interactions [2]–[4]. Expansion of htt's polyQ stretch (>37Q) causes Huntington's disease (HD), a neurodegenerative disorder characterized by the appearance of cytoplasmic (neuropil) and nuclear aggregates of mutant htt, and selective cell death in the striatum and cortex [5]–[9]. Although the mechanism of pathogenesis is still unclear, HD is recognized as a toxic gain-of-function disease, where the expansion of the polyQ stretch within htt confers new deleterious functions on the protein. The extent to which the polyQ expansion affects normal htt function is also unclear, although there is accumulating evidence that loss of normal htt function likely contributes to HD pathogenesis [10]. The polyQ stretch is conserved in vertebrate htt, and its non-pathogenic size varies from 4Q in fish, to 37Q in humans [11]–[13]. However, the polyQ stretch is absent in *Ciona* and *Drosophila* htt, and present as only a short hydrophilic NHQQ stretch in sea urchin htt, suggesting that addition of a htt polyQ stretch may be a late evolutionary feature acquired sometime after protostome-deuterostome divergence [14].

In lymphoblastoid cell lines derived from HD patients, polyQ length (in both the normal and mutant htt alleles) affects energy status, with a longer polyQ stretch correlating with a reduced cellular ATP/ADP ratio [15]. Deletion of the normal short polyQ stretch (7Q) in mouse htt (ΔQ-htt) also results in elevated ATP levels in fibroblasts derived from embryonic and adult *Hdh^ΔQ/ΔQ^* mice [16]. In addition, adult *Hdh^ΔQ/ΔQ^* mice exhibit subtly enhanced performance on the rotarod, and altered behavior in the Barnes maze learning and memory test.

To assess ΔQ-htt function in the presence of expanded polyQ htt expression, we generated mice expressing both ΔQ-htt and 140Q-htt (*Hdh^140Q/ΔQ^*). We found that ΔQ-htt expression in the HD mouse model rescued behavioral/motor deficits, reduced the number of neuropil htt aggregates, normalized brain lipofuscin levels, and enhanced lifespan relative to the HD mouse model. Clearance of htt aggregates and the accumulation of lipofuscin are mediated by autophagy, a catabolic pathway that encompasses several distinct processes in mammalian cells [17]. Macroautophagy generally involves the non-selective turnover of bulk cytoplasmic contents, including organelles and aggregated protein, and is an essential pathway for the survival of organisms during nutrient deprivation [18]. Upregulation of autophagy reduces truncated mutant htt aggregation and toxicity in both in vitro and in vivo models [19]–[22], and recently, the acetylation of soluble full-length htt has also been reported to assist its recognition by the autophagic apparatus [23]. In *Hdh^ΔQ/+^* and *Hdh^140Q/ΔQ^* mice, we observed enhanced microtubule-associated protein 1 light chain 3 (LC3, [24]) immunostaining, and increased levels of the LC3-II autophagic marker. Expression of ΔQ-htt, but not wild-type htt, induced the formation of autophagosomes in SK-N-SH neuroblastoma cells, and enhanced the clearance of truncated 74Q-htt aggregates in an autophagy-dependent process. Based on our observations, we hypothesize that deletion of the polyQ stretch within huntingtin enhances neuronal macroautophagy resulting in the more efficient clearance of neuropil mutant htt and phenotypic rescue in *Hdh^140Q/ΔQ^* mice. Moreover, we have observed that mice homozygous for ΔQ-htt expression live significantly longer than wild-type mice, an observation that is compatible with the view that enhancing constitutive autophagy may also be beneficial in normal ageing.

## Results

### Rescue of *Hdh^140Q/+^* motor and behavioral deficits in *Hdh^140Q/ΔQ^* mice

**Figure 1:**
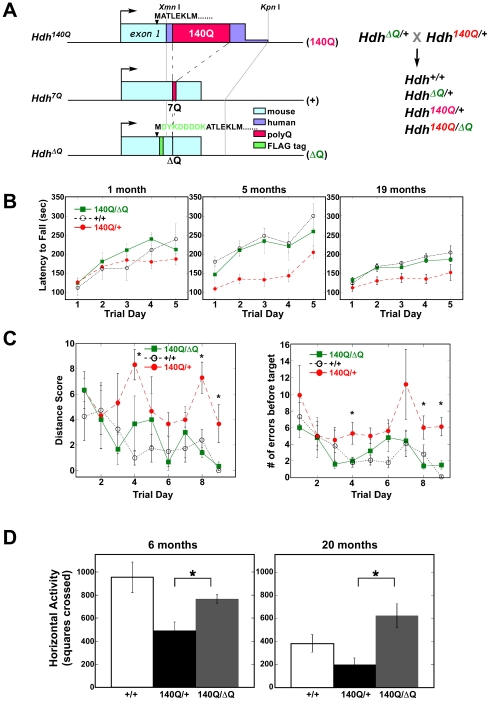
ΔQ-htt expression ameliorates motor and behavioral deficits in *Hdh^140Q/ΔQ^* mice. (A) Diagram of the *Hdh^140Q^*, wild-type (*Hdh^7Q^*), and *Hdh^ΔQ^* exon 1, and a schematic of the breeding scheme used to generate the mice employed in this study. The expansion of the polyQ stretch in the *Hdh^140Q^* allele also includes human exon 1 sequences (purple) between a conserved *Xmn*I restriction site within exon 1 and a *Kpn*I restriction site located within intron 1, while the deletion of the polyQ stretch in the *Hdh^ΔQ^* allele also includes the insertion of a FLAG epitope tag (green) following the initiation codon. Endogenous mouse sequence is shown in light blue. (B) Accelerated rotarod testing of wild-type (+/+), *Hdh^140Q/+^* (140Q/+), and *Hdh^140Q/ΔQ^* (140Q/ΔQ) mice (n = 6 for each genotype). *Hdh^140Q/+^* versus +/+ at 5 months, ANOVA for genotype; *P*<0.03 and at 19 months; *P*<0.04. The performance of the *Hdh^140Q/ΔQ^* mice did not differ significantly from the wild-type controls. (C) Barnes maze testing of 5 month old wild-type (+/+), *Hdh^140Q/+^*, and *Hdh^140Q/ΔQ^* mice (n = 5 of each genotype). *Hdh^140Q/+^* mice performed poorly on their distance scores compared to wild-type and *Hdh^140Q/ΔQ^* mice (ANOVA for genotype; F_(2,4)_ = 5.96, *P*<0.02). *Hdh^140Q/ΔQ^* and wild type mice also made significantly fewer errors than *Hdh^140Q/+^* mice before finding the target (ANOVA for genotype; F_(2,4)_ = 25.28; *P*<0.001). *Significant differences on individual trial days between *Hdh^140Q/ΔQ^* and *Hdh^140Q/+^* mice (Holm-Sidak post hoc analysis; *P*<0.001 to 0.05). (D) Horizontal activity in a novel environment was assessed in wild-type, *Hdh^140Q/+^*, and *Hdh^140Q/ΔQ^* mice (n = 5 of each genotype) at 6 and 20 months of age. At both ages, the *Hdh^140Q/+^* mice were significantly more hypoactive than the *Hdh^140Q/ΔQ^* and wild- type mice (one way ANOVA F(2,12) = 6.63, *P*<0.02 and Bonferroni post-hoc analysis, *P*<0.02 at 6 months; one-way ANOVA (F(2,14) = 6.78, *P*<0.02 and Bonferroni post-hoc analysis *Hdh^140Q/ΔQ^* versus *Hdh^140Q/+^* at 20 months, *P*<0.01).

To evaluate the impact of expressing a version of wild-type htt lacking its short polyQ stretch on the motor and behavioral phenotypes exhibited by a mouse model for HD, *Hdh^ΔQ/+^* mice were crossed with the CAG140 knock-in mouse expressing full-length htt with a chimeric human/mouse htt exon 1 containing an expanded stretch of 140 glutamines [25], (for a diagram of the knockin alleles used in this study, see Figure 1A). *Hdh^140Q/ΔQ^*, *Hdh^140Q/+^*, and wild-type control littermates were assessed using the accelerating rotarod, the Barnes maze, and an activity cage. Mice were tested on an accelerating rotating rod at 1, 5, and 19 months of age (Figure 1B). At one month of age, there were no significant differences between the wild-type controls, *Hdh^140Q/+^* mice, and the *Hdh^140Q/ΔQ^* mice (n = 6 for each genotype at 1 and 5 months, n = 4 of each genotype at 19 months). A two-way repeated measures ANOVA showed no significant effect of genotype (F_(2,6)_ = 0.87; *P*>0.05), although there was a significant trial day effect (F_(4,6)_ = 13.00; *P*<0.001), indicating that all mice were learning to stay on the rod.

At five months of age, however, the *Hdh^140Q/+^* mice performed poorly in comparison to both the wild-type control group and the *Hdh^140Q/ΔQ^* group (genotype effect; F_(2,6)_ = 5.4; *P*<0.03). Interestingly, at five months, the *Hdh^140Q/ΔQ^* mice were indistinguishable from the wild-type controls. At 19 months of age, both the wild type and *Hdh^140Q/ΔQ^* mice still performed better than the *Hdh^140Q/+^* mice and were indistinguishable from each other (genotype effect; F_(2,4)_ = 6.5; *P*<0.04), although all mice were performing more poorly at 19 months relative to their performance at 5 months of age.

At five months of age, the mice were also tested on the Barnes maze, a measure of spatial learning and memory [26]. Wild-type mice produced better scores on the Barnes maze distance test than *Hdh^140Q/+^* mice, but did not differ significantly from the *Hdh^140Q/ΔQ^* mice (n = 5 of each genotype) (Figure 1C). The distance score measures how effectively the mice are using spatial cues to locate the escape tunnel. A two way repeated measures ANOVA revealed a significant effect of genotype (F_(2,4)_ = 5.96; *P*<0.02) and a significant effect of trial day (F_(8,4)_ = 2.2; *P*<0.04). In addition, wild-type and *Hdh^140Q/ΔQ^* mice made fewer errors than *Hdh^140Q/+^* mice before finding the Barnes maze target (Figure 1C). A two-way repeated measures ANOVA revealed a significant effect of genotype (F_(2,4)_ = 25.28; *P*<0.001), and a significant effect of trial day [(F_(8,4)_ = 3.33; *P*<0.003)].

At 6 and 20 months of age, the mice were also tested in an activity cage (n = 5 of each genotype) (Figure 1D). Previous analyses of *Hdh^140Q^* mice revealed that they exhibit a period of hyperactivity, followed by hypoactivity when tested at night in an activity cage [25]. Based on total horizontal activity, the *Hdh^140Q/+^* mice were more hypoactive at night than the wild-type mice at 6 months, but the exploratory activity of the *Hdh^140Q/ΔQ^* mice did not differ significantly from wild-type controls (one-way ANOVA F_(2,12)_ = 6.63; *P*<0.02; post-hoc analysis wild-type versus *Hdh^140Q/+^*, *P*<0.02). At 20 months of age, one-way ANOVA revealed an overall difference in activity levels as well (F_(2,14)_ = 6.78; *P*<0.02). Bonferroni post-hoc analysis showed the *Hdh^140Q/+^* mice to be significantly hypoactive when compared to the *Hdh^140Q/ΔQ^* mice (*P*<0.01).

**Figure 2:**
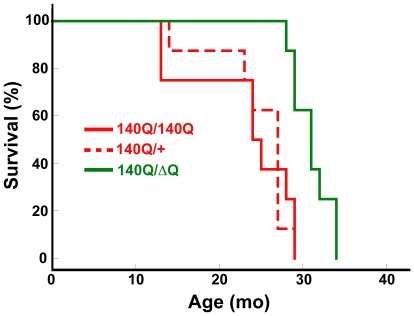
*Hdh^ΔQ^* expression enhances longevity in a knockin mouse model for HD. Kaplan-Meier survival curves are shown for *Hdh^140Q/+^*, *Hdh^140Q/140Q^*, and *Hdh^140Q/ΔQ^* mice (n = 8 mice of each genotype). The *Hdh^140Q/ΔQ^* mice lived significantly longer than either the *Hdh^140Q/140Q^* mice or the *Hdh^140Q/+^* mice (log-rank test; χ^2^ = 9.9, *P*<0.003 and χ^2^ = 11.7, *P*<0.002, respectively). The lifespans of the *Hdh^140Q/+^* and *Hdh^140Q/140Q^* mice did not differ significantly (χ^2^ = 0.03, *P* = 0.958).

*Hdh^140Q/ΔQ^* mice also exhibited a significant increase in their lifespan (median age of 31+/−0.8 months) in comparison to either *Hdh^140Q/+^* or *Hdh^140Q/140Q^* mice (median ages of 24+/−2.3 and 27+/−1.7 months, respectively, *Hdh^140Q/+^* versus *Hdh^140Q/ΔQ^* log-rank test, χ^2^ = 11.7, *P*<0.002; *Hdh^140Q/140Q^* versus *Hdh^140Q/ΔQ^* log-rank test, χ^2^ = 9.9, *P*<0.003 for n = 8 females of each genotype) (Figure 2). However, we could not detect any significant difference in the lifespan of the *Hdh^140Q/+^* and *Hdh^140Q/140Q^* mice (log-rank test, χ^2^ = 0.03, *P* = 0.958).

### Reduction of neuropil htt aggregates in *Hdh^140Q/ΔQ^* mice

**Figure 3:**
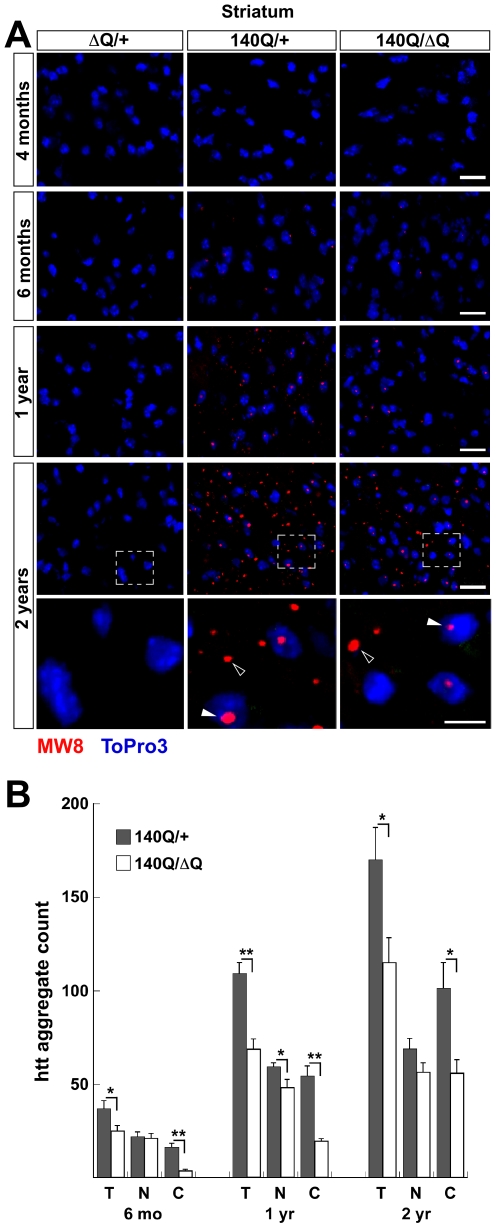
Reduced htt neuropil aggregates in the *Hdh^140Q/ΔQ^* striatum. (A) Representative confocal images of the striatum from *Hdh^ΔQ/+^*, *Hdh^140Q/+^*, and *Hdh^140Q/ΔQ^* mice at 4 months, 6 months, 1 year, and 2 years of age that were immunostained with an antibody recognizing htt aggregates (MW8, red signal). Nuclei were stained with To-Pro-3 (blue). Enlarged images of the areas enclosed by dashed white boxes are shown in the bottom panels. Open and solid white arrowheads indicate neuropil and nuclear aggregates, respectively. Scale bars = 25 µm (top panels), 10 µm (bottom three panels). (B) Total; T, nuclear; N, and neuropil; C, htt aggregate numbers from the *Hdh^140Q/+^* and *Hdh^140Q/ΔQ^* striatum (n = 4 mice of each genotype). The aggregate numbers (mean ± s.e.m.) represent counts/field from 8 images of the ventral and lateral striatum from each mouse. *P<0.05, **P<0.001.

To determine if the rescue of behavioral phenotypes in the *Hdh^140Q/ΔQ^* mice correlated with a change in the number and distribution of htt aggregates, we examined *Hdh^140Q/+^*, *Hdh^140Q/ΔQ^*, and *Hdh^ΔQ/+^* (control) brains (n = 4 of each genotype) using an antibody recognizing aggregated mutant htt in inclusions (MW8 [27]) (Figure 3A). At 4 months of age, we were unable to detect htt aggregates in either *Hdh^140Q/+^* or *Hdh^ı4̃Q̃ΔQ^* mice. Starting at 6 months of age, however, we observed a small, but similar number of nuclear aggregates in the striatum of both genotypes. In contrast, there was a significant reduction in the number of striatal neuropil aggregates observed at 6 months of age in the *Hdh^140Q/ΔQ^* brain in comparison to the *Hdh^140Q/+^* brain, *P*<0.001 (Figure 3B). At 1 year and 2 years of age, the aggregate load increases dramatically in the *Hdh^140Q/+^* brain, with the number of striatal neuropil aggregates growing more quickly with age than the number of nuclear aggregates (Figure 3B). The significant reduction in the number of striatal neuropil aggregates that was observed at 6 months of age in the *Hdh^140Q/ΔQ^* striatum was also observed in the striatum of *Hdh^140Q/ΔQ^* mice at 1 year and 2 years of age, *P*<0.001 and *P*<0.05, respectively. In the cortex, a similar marked decrease in *Hdh^140Q/ΔQ^* neuropil aggregates was observed at 6 months, 1 year, and 2 years of age (*P*<0.001–*P*<0.005) (Figure S1). In both striatum and cortex, nuclear aggregates were also reduced significantly at 1 year of age, but the magnitude of the decrease was less than that observed for the neuropil aggregates.

### Lipofuscin deposits are reduced in *Hdh^140Q/ΔQ^* mice

Increased lipofuscin has been observed in the HD brain and in the R6/2 transgenic mouse model for HD [28]–[30]. Accumulating in the lysosomes of neurons and other post-mitotic cells, lipofuscin is a yellowish-brown autofluorescent aging pigment that is composed of oxidized lipid and aldehyde cross-linked protein [31]. Lipofuscin is believed to be the byproduct of the incomplete autophagic catabolism of cellular organelles, such as mitochondria that are rich in iron. Iron and peroxide-catalyzed oxidation of incompletely digested lipid and protein results in the slow accumulation of lipofuscin in autolysosomes at a rate that correlates with metabolic activity and age of the organism [32]. In HD, oxidative stress may enhance the formation of lipofuscin, resulting in the appearance of large perinuclear lipofuscin deposits in neurons. In aged cells with high levels of lipofuscin, autophagy is diminished [33],[34], and in *C. elegans*, lower levels of lipofuscin in age-matched worms correlated with greater motility, suggesting that lipofuscin accumulation reflects biological versus chronological age [35].

**Figure 4:**
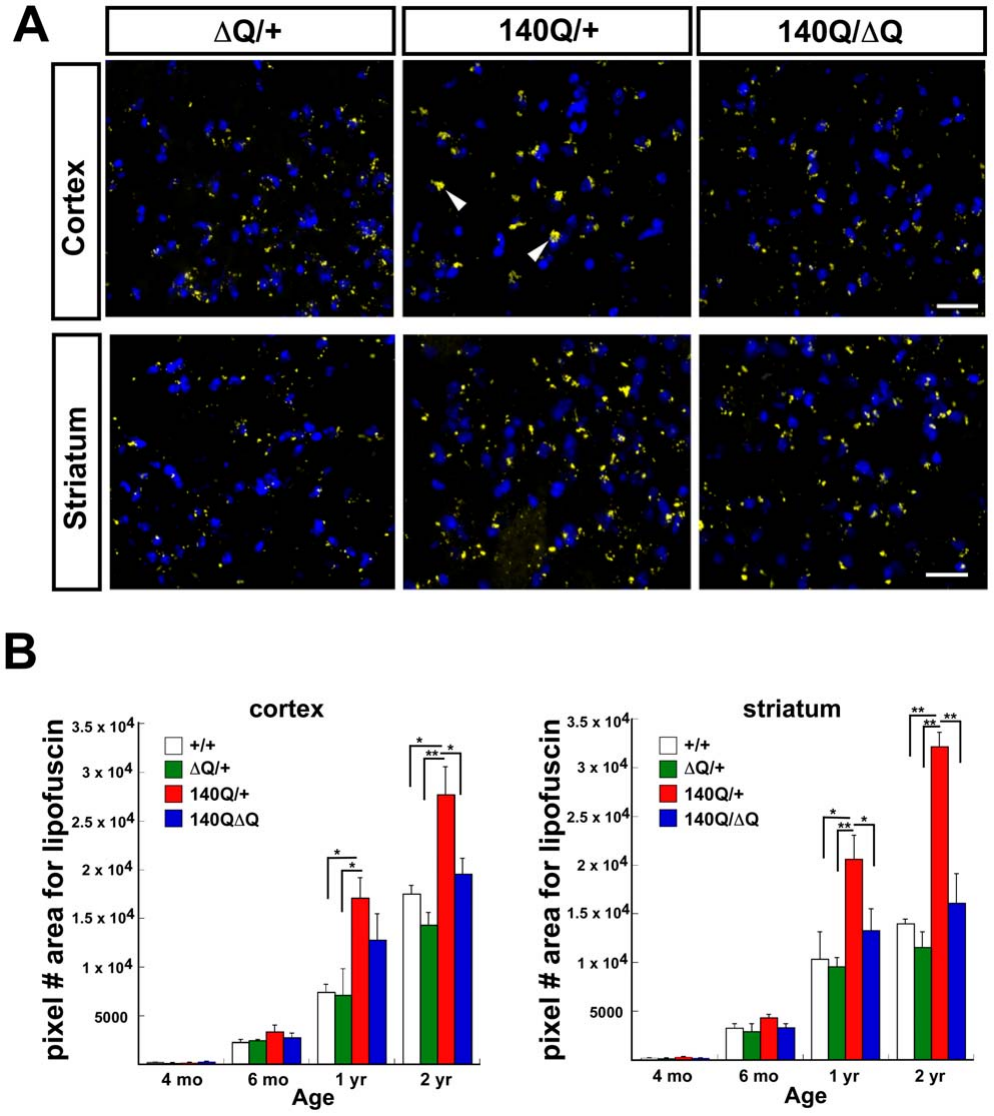
ΔQ-htt expression in *Hdh^140Q/ΔQ^* mice normalizes the increased lipofuscin accumulation that is observed in the *Hdh^140Q/+^* brain. (A) Confocal images of the parietal cortex and striatum from one year old *Hdh^ΔQ/+^*, *Hdh^140Q/+^*, and *Hdh^140Q/ΔQ^* mice (n = 4 of each genotype). Lipofuscin deposits are yellow (white arrowheads), and nuclei are stained with To-Pro-3 (blue). Scale bar = 25 µm. (B) Lipofuscin deposits at 4 months, 6 months, 1 year, and 2 years of age in wild-type (+/+), *Hdh^ΔQ/+^*, *Hdh^140Q/+^*, and *Hdh^140Q/ΔQ^* parietal cortex and striatum were quantified by measuring their pixel area/field (mean ± s.e.m.). *P<0.05, **P<0.001.

We compared the extent of lipofuscin accumulation in the striatum and cortex of wild-type, *Hdh^ΔQ/+^*, *Hdh^140Q/+^*, and *Hdh^140Q/ΔQ^* mice at 4 months, 6 months, 1 year, and 2 years of age (n = 4 mice of each genotype) (Figure 4A and 4B). Consistent with prior observations in the R6/2 HD transgenic mouse model and in postmortem HD brain tissue, we observed a significant increase in lipofuscin (measured as the pixel area of deposits in confocal images) in the striatum and cortex of *Hdh^140Q/+^* mice as they aged in comparison to wild-type mice, *P*<0.05 to *P*<0.001 (Figure 4B). Lipofuscin accumulation was greater in the striatum, relative to the cortex in the *Hdh^140Q/+^* brain. In both the *Hdh^140Q/ΔQ^* cortex and striatum, however, neuronal lipofuscin accumulation was similar to that observed in wild type controls at all ages examined.

### Altered autophagy in *Hdh^ΔQ/+^* and *Hdh^140Q/ΔQ^* mice

To determine if clearance of the neuropil htt aggregates and the reduction in lipofuscin in the *Hdh^140Q/ΔQ^* brain may be related to altered autophagy, we performed immunohistochemical analyses and western blot analyses of cellular fractions obtained from wild-type, *Hdh^ΔQ/+^*, *Hdh^140Q/+^*, and *Hdh^140Q/ΔQ^* whole brains and dissected brain regions, respectively, using an antibody to LC3. LC3 is encoded by the mammalian homolog of the yeast Atg8 gene, and is widely used as a marker for autophagy in mammalian cells because it associates tightly with autophagic membranes beginning at vesicle nucleation, and ending with its turnover in autolysosomes [24]. Western blotting with antibodies recognizing the N-terminus of LC3 detects two species with apparent molecular weights of 18 kD (LC3-I) and 16 kD (LC3-II). LC3 is processed proteolytically at its C terminus to form cytosolic LC3-I, which is conjugated to phosphatidylethanolamine on autophagosome membranes to form LC3-II. LC3-II associates specifically with autophagosome and autolysosome membranes, and LC3 vesicle numbers or levels of LC3-II correlate with autophagosome numbers [24],[36].

**Figure 5:**
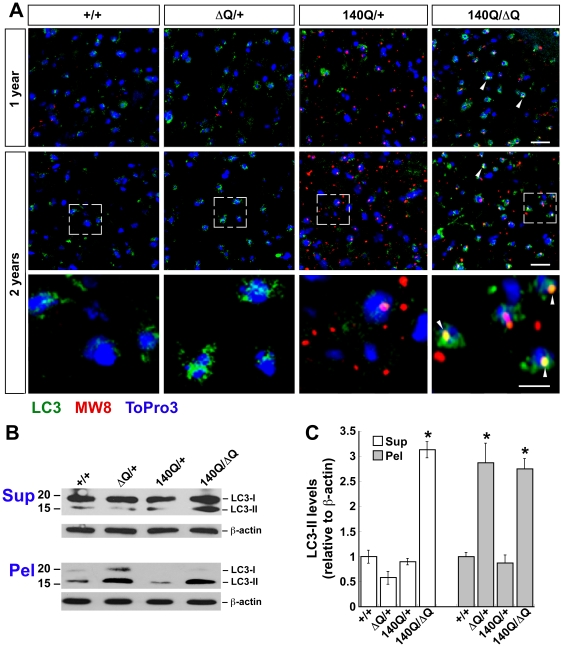
ΔQ-htt expression in mice enhances LC3 immunostaining and LC3-II steady-state levels. (A) Confocal images of LC3 (green) and htt aggregate (MW8, red) immunostaining in the striatum from 1 year and 2 year old wild-type (+/+), *Hdh^ΔQ/+^*, *Hdh^140Q/+^*, and *Hdh^140Q/ΔQ^* mice (n = 4 of each genotype). Nuclei were stained with To-Pro-3 (blue). Enlarged images of the areas enclosed by dashed white boxes are shown in the bottom panels. White arrowheads indicate examples of htt aggregates co-immunostaining with LC3 (yellow signal). Scale bars = 25 µm (top panels), 10 µm (bottom panels). (B) Western analyses for LC3-I and LC3-II in striatal supernatant (Sup) and pellet (Pel) factions from 2 year old wild-type (+/+), *Hdh^ΔQ/+^*, *Hdh^140Q/+^*, and *Hdh^140Q/ΔQ^* mice (n = 4 of each genotype). Blots were stripped and re-probed with an antibody against β-actin to control for protein loading. The positions of protein standards (in kD) are indicated on the left. (C) Quantification of LC3-II levels in the supernatant and pellet fractions relative to actin. *P<0.004 versus +/+.

LC3 immunostaining was enhanced in the striatum of *Hdh^140Q/ΔQ^* mice beginning at 6 months of age in comparison to age-matched wild-type, *Hdh^ΔQ/+^*, and *Hdh^140Q/+^* mice (n = 4 of each genotype, Figure S2). At 1 year of age, the *Hdh^140Q/ΔQ^* striatum continued to exhibit enhanced LC3 immunostaining, and at 2 years of age, elevated LC3 immunostaining was now detected in both the *Hdh^ΔQ/+^* and *Hdh^140Q/ΔQ^* striatum (Figure 5A). In contrast, LC3 immunostaining in the *Hdh^140Q/+^* striatum at 1 year and 2 years of age was not increased substantially in comparison to age-matched wild-type controls. Moreover, co-localization of LC3 immunostaining with neuropil htt aggregates was observed in the *Hdh^140Q/ΔQ^* striatum at 1 and 2 years of age, but was difficult to detect in the *Hdh^140Q/+^* striatum (Figure 5A).

To confirm that the enhanced LC3 immunostaining observed in the *Hdh^ΔQ/+^* and *Hdh^140Q/ΔQ^* striatum was due to an increase in LC3-II levels, dissected striata from 2 year old wild-type, *Hdh^ΔQ/+^*, *Hdh^140Q/+^*, and *Hdh^140Q/ΔQ^* mice (n = 4 of each genotype) were homogenized and separated into supernatant (NP40-soluble) and pellet (NP40-insoluble) fractions, and then analyzed by western blotting with an antibody that recognizes both LC3-I and LC3-II (Figure 5B and 5C). In the soluble protein fractions, an increase in LC3-II was observed in the *Hdh^140Q/ΔQ^* striatum. Interestingly, LC3-II was also enriched in the striatal pellet fractions from both *Hdh^ΔQ/+^* and *Hdh^140Q/ΔQ^* mice. In contrast, LC3-II was present at only low levels in the wild-type and *Hdh^140Q/+^* pellet fractions. A corresponding western blot analysis of LC3 levels in total (unfractionated ) protein extracts from 2 year old mice revealed an increase in LC3-II in both the *Hdh^ΔQ/+^* and *Hdh^140Q/ΔQ^* samples (Figure S3B). We note that we also observed an enrichment of both the autophagy protein beclin 1 and lysosome-associated membrane protein type 1 (Lamp1) levels in the 800×g low-speed P1 fractions from *Hdh^ΔQ/+^* and *Hdh^140Q/ΔQ^* striatal extracts prepared by lysis in the absence of detergent (Figure S3A). Overall levels of beclin 1 and Lamp1 in total brain extract, however, were similar in all genotypes (Figure S3B). Lamp1 is a marker for late endososomes, amphisomomes (formed after autophgagosome-late-endosome fusion), dense autolysosomes and lysosomes that are enriched in the 800×g P1 fraction [37], and these observations, together with our findings related to the alterations in beclin 1 and LC3-II fractionation, suggest that the subcellular distribution of several components of the autophagy pathway are altered by ΔQ-htt expression.

It was proposed recently, that htt's association with the ER via its N1–17 domain allows it function as a sensor of ER stress, and to potentially regulate autophagy [1],[38]. In previous work, we found no obvious difference in the nuclear/cytoplasmic localization of ΔQ-htt in comparison to wild-type htt in early passage wild-type and *Hdh^ΔQ/ΔQ^* primary mouse embryonic fibroblasts (PMEFs) [16]. To analyze further the subcellular localization of wild-type- and ΔQ-htt together with markers for the ER (calnexin), and to assess a marker for autophagy (LC3), we performed immunocytochemistry on passage 5 (P5) cultures of wild-type and *Hdh^ΔQ/ΔQ^* primary mouse embryonic fibroblasts (PMEFs) (Figure S4). P5 cultures of wild-type fibroblasts are actively dividing, while P5 cultures of *Hdh^ΔQ/ΔQ^* fibroblasts are, in contrast, undergoing replicative senescence [16]. Wild-type- and ΔQ-htt were detected in both the cytoplasm and nucleus, and perinuclear localization of wild-type- and ΔQ-htt with the ER marker, calnexin was also detected in both *Hdh^+/+^* and *Hdh^ΔQ/ΔQ^* PMEFs (Figure S4A, S4B). However, nuclear localization of htt appeared to be increased in those cells with a more senescent morphology (i.e. more flattened/spread appearance). Perinuclear LC3 immunoreactivity was also enhanced in the *Hdh^ΔQ/ΔQ^* PMEFs with a senescent morphology (Figure S4C), suggesting the possibility for increased autophagy in those *Hdh^ΔQ/ΔQ^* PMEFs undergoing replicative senescence.

### Expression of ΔQ-htt enhances autophagosome synthesis in vitro

**Figure 6:**
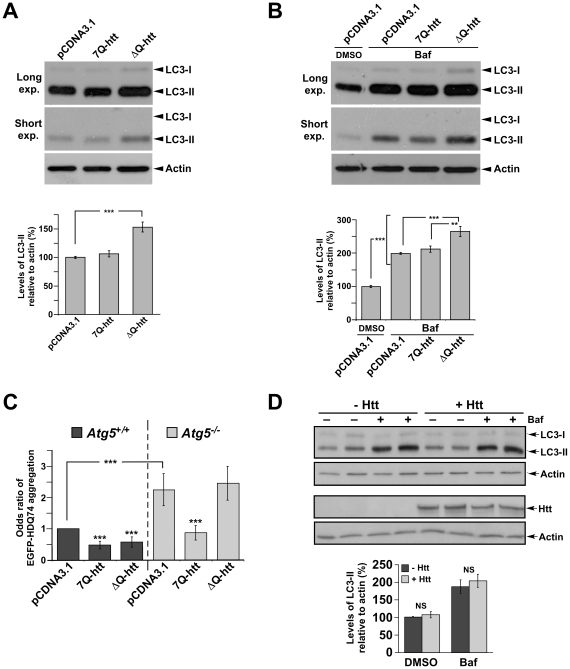
ΔQ-htt expression increases autophagosome synthesis and Atg5-dependent reduction of EGFP-74Q truncated htt aggregates in vitro. (A) SK-N-SH cells were transfected with control vector (pCDNA3.1), 7Q-htt, or ΔQ-htt constructs (transfections performed in triplicate at least twice), and cell lysates were analyzed for LC3-II levels 24 hr post-transfection by western blotting with an anti-LC3 antibody (for comparison, both longer and shorter exposures are shown), and densitometric analysis relative to actin levels. ΔQ-htt versus control; (***P<0.001), ΔQ-htt versus 7Q-htt; (*P*<0.002). (B) SK-N-SH cells were transfected with control vector, 7Q-htt, or ΔQ-htt constructs and treated with either DMSO (pCDNA3.1 transfected cells) or with 400 nM bafilomycin A_1_ (Baf) in DMSO (pCDNA3.1, 7Q-htt, or ΔQ-htt transfected cells) for 4 hours at 20 hr post-transfection. Autophagosome synthesis was analyzed by western blotting using an anti-LC3 antibody and densitometric analysis relative to actin. ***P<0.0001, **P<0.004. (C) *Atg5^+/+^* (autophagy-competent) and *Atg5^−/−^* (autophagy-deficient) mouse embryonic fibroblasts were transfected with an EGFP-HDQ74 construct together with vector control, 7Q-htt, or ΔQ-htt constructs, and then assessed for the proportion of EGFP-positive cells with EGFP-74Q-htt aggregates by calculating the odds ratio 48 h post-transfection. ***P<0.0001 for both ΔQ-htt versus control, and for 7Q-htt versus control in *Atg5^+/+^* cells, *P*<0.0001 for 7Q-htt versus control in *Atg5^−/−^* cells. ΔQ-htt's ability to reduce aggregates is abolished in *Atg5^−/−^* cells (*P* = 0.36 in comparison with control vector transfected cells). (D) *Hdh^ex4/5/ex4/5^* knock-out (*Hdh^−/−^*) mouse embryonic stem (ES) cells were transfected with EGFP along with either pCI (empty vector) or full-length wild-type huntingtin (1∶5 ratio) for 4 h, and then treated with DMSO (– Baf ) or 400 nM bafilomycin A_1_ in DMSO (+ Baf) for the last 2 h of the 48 h post-transfection period. Cells were then FACS-sorted for the EGFP-positive cells, in which LC3-II levels were assessed by western blotting and densitometric analysis relative to actin. Htt levels were assessed by western blotting using MAB2166. There were no significant changes in LC3-II levels in the huntingtin transfected *Hdh^−/−^* ES cells compared to empty vector transfected *Hdh^−/−^* ES cells in the absence (*P* = 0.7103) or presence (*P* = 0.4063) of bafilomycin A_1_.

An alteration to autophagy resulting in the increased steady-state levels of LC3-II can be attributed to either enhanced autophagic flux, or to a block in a later step within the pathway that would interfere with the turnover of LC3-II in the autolysosome [39]. To determine if ΔQ-htt can enhance autophagosome synthesis, we transfected SK-N-SH neuroblastoma cells with full-length wild-type (7Q-htt) or ΔQ-htt cDNA expression constructs (diagrams in Figure S5), and monitored the levels of LC3-II 24 h post-transfection by western blotting (Figure 6A). The levels of LC3-II were increased significantly in the ΔQ-htt transfected cells in comparison to either control vector- or 7Q-htt-transfected cells. To monitor autophagy by an alternative method, we also transfected HeLa cells with an EGFP-LC3 expression construct, together with pCDNA3.1 (vector control), 7Q-htt or ΔQ-htt in a 1∶3 ratio (Figure S6). The proportion of EGFP-positive cells with >10 EGFP-LC3 vesicles was assessed and expressed as an odds ratio with 95% confidence limits. ΔQ-htt transfection, but not 7Q-htt transfection, increased the proportion of cells with EGFP-LC3 vesicles. To measure autophagosome synthesis, the cDNA constructs were also transfected in the presence or absence of the antibiotic bafilomycin A_1_, a vacuolar H+ ATPase inhibitor that suppresses turnover of LC3-II in autolysosomes [40]–[42]. Thus, measuring the levels of LC3-II in the presence of bafilomycin A_1_ measures LC3-II formation, as the antibiotic blocks LC3-II degradation. The levels of LC3-II were increased significantly in the bafilomycin A_1_-treated and ΔQ-htt-transfected cells in comparison to the bafilomycin A_1_- treated cells alone or the bafilomycin A1-treated and 7Q-htt-transfected cells, suggesting that ΔQ- but not 7Q-htt expression results in increased autophagosome synthesis (Figure 6B). To confirm that an increase in LC3-II formation resulting from ΔQ-htt expression is enhancing autophagic activity that can remove another autophagy substrate, 7Q- or ΔQ-htt constructs were transfected into *Atg5^+/+^* (autophagy-competent) and *Atg5^−/−^* (autophagy-deficient) mouse embryonic fibroblasts [43], together with an EGFP-tagged 74Q-htt exon 1 construct (EGFP-HDQ74) expressing an N-terminal fragment of mutant htt that forms aggregates readily in vitro [44]. Aggregate formation in EGFP-positive cells 48 h post-transfection was assessed by calculating odds ratios with 95% confidence limits [44]–[46] (Figure 6C). The proportion of cells with EGFP-HDQ74 aggregates was significantly reduced in *Atg5^+/+^* cells transfected with ΔQ-htt, but not in *Atg5^−/−^* cells. Interestingly, 7Q-htt overexpression also reduced aggregate load in both *Atg5^+/+^* and in *Atg5^−/−^* cells. These data suggest that while ΔQ-htt can induce autophagic clearance of mutant htt aggregates, 7Q-htt overexpression may induce a reduction in aggregate numbers or formation via an autophagy-independent mechanism in our in vitro system. Taken altogether, these data support the hypothesis that ΔQ-htt expression can stimulate autophagosome formation and the Atg5-dependent clearance of htt aggregates.

Importantly, we saw no difference in autophagy in 7Q-htt-overexpressing cells versus empty vector transfected cells (Figure 6A and 6B), or when comparing huntingtin knockout (*Hdh^ex4/5^*/*Hdh^ex4/5^* [47]) mouse embryonic stem cells (*Hdh^−/−^*) which were either transfected with empty vector of with wild-type full-length 17Q-Htt (Figure 6D), suggesting that the ability of htt to induce autophagy is a specific consequence of the loss of its polyQ tract.

### ΔQ-htt expression stimulates autophagy via an mTOR–independent pathway

**Figure 7:**
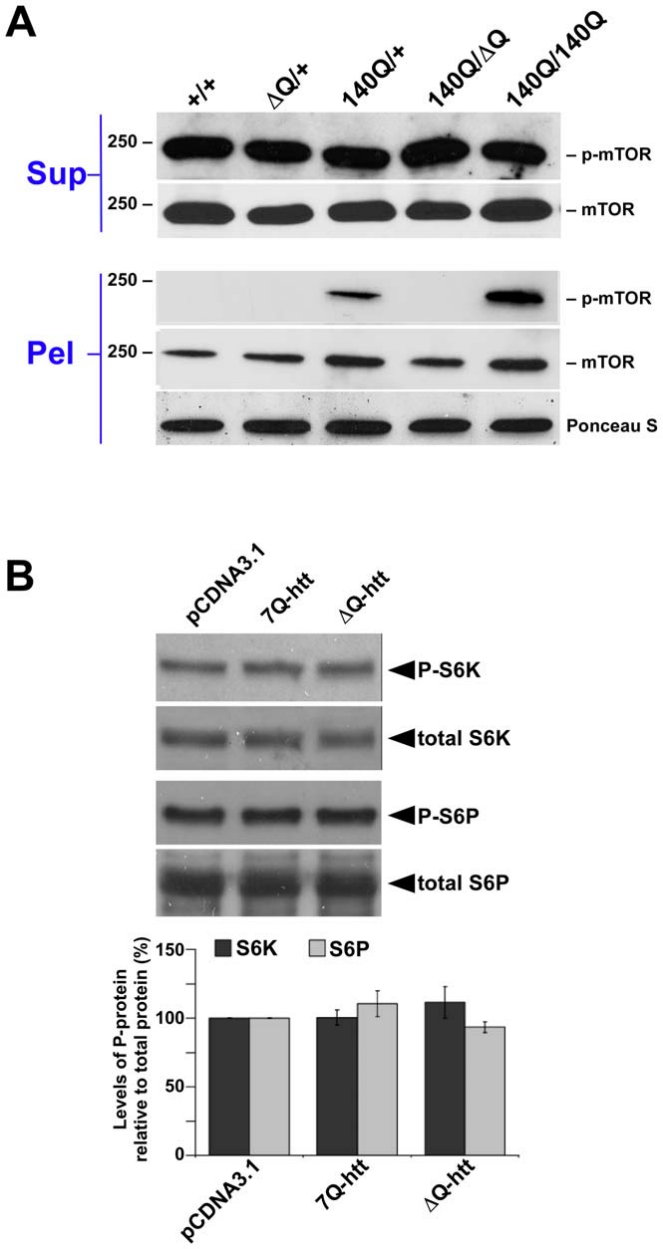
ΔQ-htt expression stimulates autophagy through an mTOR–independent mechanism. (A) Western blot analysis of the supernatant (Sup) and pellet (Pel) fractions from the striatum of 2 year old wild-type (+/+), *Hdh^ΔQ/+^*, *Hdh^140Q/+^*, *Hdh^140Q/ΔQ^*, and *Hdh^140Q/140Q^* mice probed with an antibody against phospho-mTOR (p-mTOR). The blots were stripped and re-probed with an antibody (mTOR) recognizing both phosphorylated (enzymatically active form) and non-phosphorylated mTOR (enzymatically inactive form correlating with the induction of autophagy). For protein loading control, a strip from the blot was stained with Ponceau S. The size of standards (in kD) is indicated on the left. (B) SK-N-SH cells transfected with vector control (pCDNA3.1), 7Q-htt, or ΔQ-htt constructs (transfections were performed in triplicate at least twice) were analyzed for mTOR activity 24 h post-transfection by western blotting using antibodies specific for two targets of mTOR kinase activity: S6 kinase (S6K) and S6 ribosomal protein (S6P). The relative levels of phospho-S6K (P-S6K) and phospho-S6P (P-S6P) to total S6K and S6P, respectively, were determined by densitometric scanning of western blots probed with antibodies specific for phospho- and total S6K or S6P. ΔQ-htt expression had no significant effect on the level of P-S6K (7Q-htt versus vector control, *P* = 0.96; ΔQ-htt versus vector control, *P* = 0.30) or P-S6P (7Q-htt versus vector control, *P* = 0.26; ΔQ-htt versus vector control, *P* = 0.47).

A central regulator of metabolism and autophagy in both invertebrates and vertebrates is TOR (Target of Rapamycin) kinase, and inhibition of TOR kinase activity by rapamycin and its analogs has been used successfully to stimulate autophagic clearance of mutant htt aggregates in both *Drosophila* and mouse models for HD [48]. To determine if the activity of mammalian TOR (mTOR) is inhibited by ΔQ-htt expression, we examined the phosphorylation status of mTOR in the striatum of two year old wild-type, *Hdh^ΔQ/+^*, *Hdh^140Q/+^*, *Hdh^140Q/140Q^*, and *Hdh^140Q/ΔQ^* mice, and also the phosphorylation status of downstream targets of mTOR in our in vitro system (Figure 7). Phospho-mTOR (p-mTOR) levels correlate positively with mTOR kinase activity and inversely with mTOR inhibition and the activation of macroautophagy [48], although autophagy can also be regulated by mTOR-independent pathways. We observed no difference in p-mTOR levels in the supernatant fractions of all genotypes examined (Figure 7A). However, we did detect an enrichment of p-mTOR in the striatal pellet fractions from the *Hdh^140Q/+^* and *Hdh^140Q/140Q^* brains. This association of p-mTOR with the pellet fraction likely represents p-mTOR association with htt aggregates, as was observed previously both in vitro, and in a transgenic HD mouse model [48].

To confirm our in vivo analyses, SK-N-SH cells were transfected with either 7Q- or ΔQ-htt expression constructs and the phosphorylation status of two targets of mTOR kinase activity were assessed 24 h post-transfection (Figure 7B). The levels of phospho-S6 kinase and phospho-S6 ribosomal protein were not significantly different in the cells transfected with 7Q- or ΔQ-htt, supporting the hypothesis that ΔQ-htt's upregulation of autophagy is not mediated by a reduction in mTOR kinase activity.

### ΔQ-htt expression extends lifespan in the mouse

**Figure 8:**
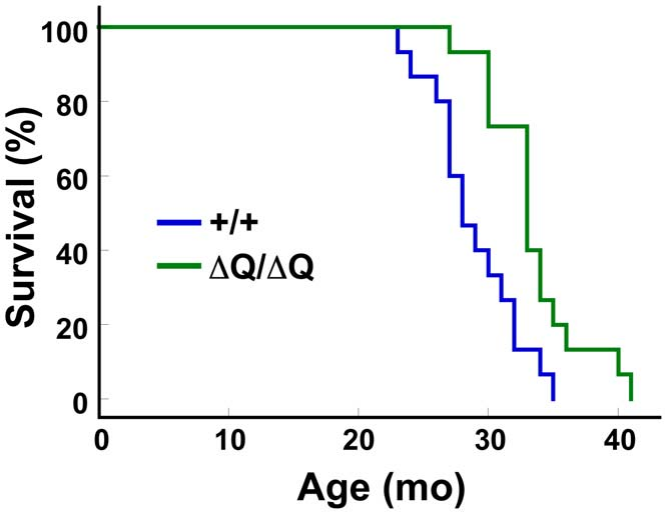
*Hdh^ΔQ/ΔQ^* mice live significantly longer than wild-type mice. Kaplan-Meier survival curves are shown for wild-type (+/+, n = 15; 1 male and 14 females) and *Hdh^ΔQ/ΔQ^* (n = 15; 2 males and 13 females) mice. A significant extension of lifespan was observed in the *Hdh^ΔQ/ΔQ^* mice in comparison to the wild-type mice (χ^2^ = 9.6, *P*<0.005; Mantel-Cox log-rank test).

Our observations suggest that expression of a version of htt lacking its normal stretch of polyQ can enhance autophagic clearance of neuropil mutant htt inclusions. During normal aging, misfolded and aggregated proteins accumulate due to an apparent decline in the function of lysosomal degradation pathways [49]. In *Caenhorhabditis elegans*, autophagy is an essential component in the mechanism that extends lifespan upon dietary restriction. Knockdown of essential autophagy genes, for example, shortens lifespan in *C. elegans*, and suppresses lifespan extension induced by dietary restriction, reduced mitochondrial function, and alterations in insulin/IGF-1 or TOR signaling [50]. Moreover, enhancing basal levels of autophagy in the nervous system of *Drosophila* by Atg8a overexpression increases both longevity and resistance to oxidative stress [51]. However, the ability of autophagy upregulation to extend mammalian lifespan has not previously been tested. To determine if ΔQ-htt expression has an effect on longevity in the absence of 140Q-htt expression, we assessed the lifespan of *Hdh^ΔQ/ΔQ^* mice in comparison to wild-type mice (n = 15 mice of each genotype). While the wild-type controls lived to 28+/−1.3 months (median age +/− s.e.m.), the *Hdh^ΔQ/ΔQ^* mice lived to a median age of 33+/−1.1 months, representing an 18% extension of lifespan (log-rank test, c^2^ = 9.6, *P*<0.005) (Figure 8). The oldest *Hdh^ΔQ/ΔQ^* mouse survived for approximately 3.5 years in our colony, compared to 3 years for the oldest wild-type mouse.

## Discussion

We provide evidence that expression of a version of mouse htt that lacks its short 7Q polyglutamine domain can stimulate the formation of autophagosomes in vitro and enhance the clearance of htt neuropil aggregates, ameliorate behavioral/motor phenotypes, and extend lifespan in a mouse model for HD. When expressed in homozygosity, ΔQ-htt can also significantly extend lifespan in the mouse. Recently, it was proposed that htt may participate directly in autophagy because of its structural similarity with mTOR, and also because it co-localizes partially with autophagosomes [38]. These results, and our data, do not address directly whether or not htt has a normal function involved in autophagy. However, our results do suggest that deletion of htt's short 7Q stretch can enhance basal autophagy, and are compatible with a hypothesis suggesting that htt's polyQ stretch may modulate a normal function for htt in this process. Expansion of htt's polyQ stretch beyond the pathogenic threshold may, in contrast, suppress such a function, and account for our observation that we did not observe autophagy induction in the *Hdh^140Q/+^* brain. In this scenario, ΔQ-htt's gain-of-function in autophagy would be dominant to a potential loss-of-function in autophagy caused by the expansion of the polyQ stretch.

The rescue of motor and behavioral phenotypes in the *Hdh^140Q/ΔQ^* mice starting at 5–6 months of age correlates with a reduction in neuropil htt aggregates, and a normalization of lipofuscin levels. Neuropil aggregates are an early phenotypic feature of HD, and our results are compatible with a recent study demonstrating that clearance of cytoplasmic htt aggregates by the expression of an intrabody specific for aggregated htt can rescue motor and behavioral deficits in a transgenic mouse model for HD [52]. The reduced number of nuclear inclusions observed in the *Hdh^140Q/ΔQ^* brain at one year of age may be due to htt's ability to shuttle between the nucleus and cytoplasm [1]. The stimulation of autophagy in other HD mouse models, for example, can also reduce htt nuclear aggregate number [48]. The normalization of lipofuscin deposits in the *Hdh^140Q/ΔQ^* brain may be the consequence of reduced oxidative stress that is secondary to the reduction in mutant htt aggregate load. Although we had previously detected increased lipofuscin in the *Hdh^ΔQ/ΔQ^* brain [16], we observed similar levels of lipofuscin in the *Hdh^ΔQ/+^* and wild-type brain. As lipofuscin is an end-product of autophagy, the increased lipofuscin accumulating in the *Hdh^ΔQ/ΔQ^* brain may represent the increased turnover of mitochondria due to the expression of two *Hdh^ΔQ^* alleles. In this case, increased lipofuscin accumulation could represent increased autophagic activity instead of increased oxidative stress.

The mitochondrial-lysosomal axis theory of postmitotic cellular ageing posits that during ageing, autophagic capacity decreases, mitochondrial turnover declines, and damaged mitochondria and protein aggregates accumulate [53]. Old or damaged mitochondria will produce less ATP and more superoxide radicals leading to increased oxidative stress. This causes a positive feedback loop resulting in further damage. There is accumulating evidence that enhanced autophagy correlates with increased longevity in *C. elegans* and *Drosophila*. In *C. elegans*, autophagy is an essential component in the mechanism that extends lifespan upon dietary restriction [50]. Knockdown of essential autophagy genes, for example, shortens lifespan in *C. elegans*, and suppresses lifespan extension induced by dietary restriction, reduced mitochondrial function, and alterations in insulin/IGF-1 or TOR signaling. In a complementary experiment, enhancing basal levels of autophagy in the nervous system of *Drosophila* by Atg8a overexpression increases both longevity and resistance to oxidative stress [51].

The 18% increase in *Hdh^ΔQ/ΔQ^* lifespan relative to wild-type controls is comparable to that observed with mouse mutations in insulin signaling pathways that result in increased longevity. Mice heterozygous for a knock-out of the insulin-like growth factor 1 receptor gene (*Igf1r*) exhibit a 26% increase in mean lifespan [54], while mice expressing a mutant insulin receptor gene in adipose tissue exhibit an 18% increase in lifespan [55]. Mice lacking expression of the insulin receptor substrate 1 gene (*Irs*1^−/−^) also live 18% longer than wild-type mice [56], and brain-specific knock-out of one *Irs2* allele in the mouse results in an 18% extension of lifespan [57]. Our findings do not permit us to determine if increased lifespan in the *Hdh^ΔQ/ΔQ^* mice is the consequence of neuronal or global expression of ΔQ-htt. Neuronal-specific overexpression of Atg8a in *Drosophila* is sufficient to extend lifespan [51], but analogous experiments in mice have not yet been performed.

The effect of ΔQ-htt expression on *Hdh^ΔQ/ΔQ^* lifespan appears, at first, to be incompatible with the premature replicative senescence phenotype that we observed in *Hdh^ΔQ/ΔQ^* PMEFs cultured in vitro [16]. However, the detection of both increased senescence-associated (SA)-β-galactosidase staining [16],[58], and LC3 immunoreactivity (Figure S4C) in senescent PMEFs supports the data of Narita and colleagues suggesting that upregulating autophagy may facilitate the mitotic senescence transition in vitro [59]. In this scenario, ΔQ-htt expression may have opposite effects on cellular senescence and mammalian lifespan. A similar, apparently contradictory, response to mammalian SIRT1 expression (a homolog of the yeast Sir2 factor involved in extending replicative lifespan) has been described in PMEFs where absence of SIRT1 expression increases replicative lifespan [60]. Thus, in these examples, replicative lifespan in vitro may not always correlate positively with organismal lifespan.

Upregulation of autophagy has the potential to be a therapeutic strategy for Huntington's disease and related disorders. Although rapamycin and its analogs have proven to be very useful in stimulating increased clearance of N-terminal truncated mutant htt aggregates in various animal models for HD via a “pulsitile” upregulation of autophagy, our data suggest that tonic long-term autophagy upregulation via ΔQ-htt expression is not associated with overt side-effects. This genetic method for autophagy upregulation is apparently mTOR independent based on our inability to detect a significant decrease in soluble p-mTOR levels in vivo, and alterations in the phosphorylation status of downstream mTOR kinase targets in vitro. In this regard, we have identified a series of novel compounds that influence autophagy in an mTOR-independent fashion [45],[61]. Although further work is required to elucidate the pathway responsible for ΔQ-htt's affect on autophagy, our findings support the view that the development of both genetic and small molecule-based therapeutic strategies aimed at stimulating the autophagic clearance of aggregated protein may be of use in both the treatment of neurodegenerative disease, and in lifespan extension.

## Methods

### Generation of mice

*Hdh^+/+^*, *Hdh^ΔQ/+^*, *Hdh^140Q/+^*, and *Hdh^140Q/ΔQ^* mice were obtained from heterozygous intercrosses between *Hdh^ΔQ/+^* and *Hdh^140Q/+^* mice that were maintained in a mixed 129/Sv and C57BL6 background. *Hdh^ΔQ/ΔQ^* and *Hdh^140Q/140Q^* mice were obtained from *Hdh^ΔQ/+^* and *Hdh^140Q/+^* intercrosses, respectively. All protocols for animal use were approved by the Institutional Animal Care and Use Committee of the University of Virginia, and were in accordance with NIH guidelines. For routine genotyping, PCR was used to confirm the presence of the different *Hdh* alleles: ΔQ allele; ΔQ-for = 5′-GACGGGCCCAAGATGG-3′ and ΔQ-rev = 5′-GGCGGTGGAAACGACTT-3′ amplify a 226 bp product from only the ΔQ allele, while Epi-for = 5′-GCGTAGTGCCAGTAGGCTCCAAG-3′and Epi-rev = 5′-CTGAAACGACTTGAGCGACTCGAAAG-3′ flank the site of the FLAG epitope in the ΔQ allele and amplify either a 112 bp product from the wild-type allele or a 136 bp product from the ΔQ allele. 140Q allele; 140-for = 5′-CTGCACCGACCGTGAGTCC-3′and 140-rev = 5′-GAAGGCACTGGAGTCGTGAC-3′ flank a small intron-1 deletion created during the generation of the 140Q allele. A wild-type allele (or ΔQ allele) will generate a 235 bp product, while the 140Q allele will generate a 150 bp product. To verify that the mean CAG repeat length in the *Hdh^140Q^* allele was similar in the *Hdh^140Q/+^* and *Hdh^140Q/ΔQ^* mice that were used for our analyses, the CAG repeat was amplified using CAG-1 = 5′-CTTCGAGTCCCTCAAGTCCTTC-3′and CAG-2 = 5′-GGTGGCGGCTGTTGCTGCTG-3′ (data not shown). These oligonucleotides are specific for the human sequence surrounding the CAG repeat in the *Hdh^140Q^* allele and will generate a ∼450 bp product using the Expand High Fidelity PCR system (Roche Molecular Diagnostics).

### Motor and behavioral analyses

The accelerating rotarod test was performed at 1, 5, and 19 months of age (n = 6 mice of each genotype at 1 and 5 months of age, and n = 4 mice of each genotype at 19 months of age; all mice in the same cohort) as described [16]. At each time-point, there were 3 separate testing sessions of 5 days (3 trials per day) to control for environmental factors. The Barnes maze testing was performed at 5 months of age according to methods described previously (n = 5 mice of each genotype) [16]. There were 3 separate testing sessions of 9 days to control for environmental factors. Activity testing was performed at 6 and 20 months of age (separate cohorts) according to methods described previously, except that tests were performed between the hours of 7 pm - 6 am, as open field activity is dependent upon the resting state of the mouse, with more activity anticipated during nocturnal hours [25]. There were 3 separate testing sessions with a mix of genotypes in each session.

### Immunohistochemical and immunocytochemical analyses

Rapidly frozen mouse brains were sectioned at 14 µm using a cryostat (Bright Instrument Co.). Sections were washed briefly in PBS, fixed for 10 min in 4% paraformaldehyde in 0.1M phosphate buffer pH 7.4 or in 4% paraformaldehyde for 10 min, followed by rinse in PBS and a second fixation step in 100% methanol for 15 min on ice (both conditions yielded identical results, data not shown). Sections were washed in PBS before blocking with 5% donkey serum, 0.1% Triton ×100, in PBS for 1 h at RT, and then incubated o/n at 4°C with primary antibody diluted in 5% donkey serum, 0.1% Triton ×100 in PBS. Primary antibodies used were: rabbit polyclonal LC3 (1∶100, Novus Biologicals), and mouse monoclonal MW8 (1∶70, Developmental Studies Hybridoma Bank). Following the primary antibody incubation, sections were washed in PBS three times and incubated with secondary antibody (donkey anti-mouse, rabbit or guinea pig-Cy3 or –FITC, Jackson Immunologicals) together with the fluorescent DNA stain To-Pro-3 iodide (Invitrogen) for 1 h at RT. Sections were then washed with PBS before treatment to suppress lipofuscin autofluorescence by incubating sections sequentially in 75% ethanol for 5 min, lipofuscin eliminator reagent (Chemicon/Millipore) for 5 min, and 5 min in 75% ethanol. Sections were then mounted with Vectashield (Vector Laboratory), and examined using a Nikon C1-confocal microscope.

For immunocytochemical analyses, PMEFs were seeded at a concentration of 1×10^4^ cells/ml onto 4-well chamber slides (Nunc). Two days following plating, the cells were washed briefly two times with PBS, fixed in 4% paraformaldehyde in 0.1 M phosphate buffer pH 7.4 for 10 min at RT followed with a 10 min incubation in cooled 100% methanol on ice, and then washed three times for 5 min at RT in PBS. The cells were blocked in PBS containing 5% donkey serum, 0.1% Triton X-100, and donkey anti-mouse IgG FAB (1∶400) for 1 h at RT. The cells were then washed three times in PBS (10 min each wash at RT), and then blocked again in PBS containing 5% donkey serum, 0.1% Triton X-100 for 1.5 h at RT. The cells were incubated with primary antibody diluted in blocking solution for 2 h at RT, and then washed three times for 5 min at RT in PBS. The cells were then incubated with secondary antibody diluted in blocking solution, and then washed again three times for 5 min each at RT in PBS. Slides were then immersed in 70% ethanol for 5 min and then treated with 1 drop of autoflorescence eliminator reagent (Millipore/Chemicon) for an additional 5 min. Slides were coverslipped in aqueous mounting medium and imaged using an Olympus BX51 microscope equipped with an Olympus MagnaFire CCD camera. Primary antibodies used were: goat anti-Calnexin (C-20), Santa Cruz Biotechnology, 1∶200; mouse anti-LC3 (5F10), Nanotools, 1∶100; mouse anti-FLAG M2 (F3165), Sigma, 1∶100; and mouse anti-htt MAB2166, Millipore, 1∶100.

### Quantification of htt aggregates and EGFP-LC3 vesicles

#### Tissue sections

Sections were imaged with a 60× objective using a Nikon C1 confocal microscope, and the numbers of protein aggregates were counted manually (blind to genotype) from 14 µm coronal and sagittal sections through the striatum. Neuropil and nuclear inclusions were counted separately. A z-stack was performed using the confocal microscope to confirm the presence of MW8-positive aggregates in both the cytoplasm and the nucleus (data not shown). Htt aggregates in the cortex from the same sections used to obtain the striatal htt aggregates counts were also quantified.

#### Cell culture analyses

The percentage of EGFP-positive cells with EGFP-HDQ74 aggregates was determined as previously described [44]–[46]. Analysis and acquisition of images were done with a Nikon Eclipse E600 fluorescence microscope (plan-apo 60×/1.4 oil immersion lens). Quantification of cells with EGFP-LC3 vesicles was performed as described previously [45].

### Lipofuscin imaging and quantification

Lipofuscin accumulation analyses were performed using 14 µm fresh frozen brain sections. Sections were fixed for 15 min on ice in 100% methanol, washed in PBS, and then incubated with To-Pro-3 iodide (1∶10,000 dilution in PBS) for 1 h at RT. Sections were then washed in PBS and mounted using Vectashield. Confocal images were acquired in the green and red channels (lipofuscin has a broad autofluorescent emission spectrum from 500 nm to 650 nm). Yellow pixel areas corresponding to the lipofuscin deposits were quantified using ImagePro 4.5 (Media Cybernetics) software from 8 images of the ventral striatum or parietal cortex obtained from each brain (n = 4 brains of each genotype for each age analyzed).

### Tissue fractionation

Dissected striata from an individual brain were homogenized on ice in 500 µl 50 mM Tris-HCl pH 8.5, 100 mM NaCl, 5 mM MgCl_2_, 1 mM EDTA, 0.5% NP-40 supplemented with 5 mM NaF, 1 mM Na_3_VO_4_, and a protease inhibitor mixture (Complete –EDTA tablets, Roche). The tissue homogenate (total or unfractionated sample) was then centrifuged for 10 min at 4°C at 16,100×g to obtain crude cytoplasmic (supernatant) and nuclear pellet fractions. The pellet was suspended by dounce homogenization in 100 µl homogenization buffer, and incubated with 0.2 mg/ml final concentration of DNAse I for 60 min on ice. The suspension was then centrifuged for 10 min at 4°C at 16,100×g to obtain the final pellet fraction. The pellet was resuspended in 100 µl homogenization buffer, and the protein concentration in the supernatant and pellet samples was determined using the BCA assay (Pierce). Typically, there was a 20-fold excess of protein recovered from the supernatant fraction relative to the pellet fraction. 30 µg of each fraction was analyzed by western blotting. Although some htt N-terminal fragments can be solubilized by SDS-PAGE sample buffer extraction of the pellet fraction, the majority of htt material in the pellet consists of aggregates (Figure S7). For the generation of a P1 low-speed pellet fraction (Figure S3A), striatal tissue from each brain was dounce-homogenized on ice in 500 µl 15 mM Tris-HCl pH 7.6, 0.25 M sucrose, 1 mM MgCl_2_, 2.5 mM EDTA, 1 mM EGTA, 1 mM DTT, 5 mM NaF, 1 mM Na_3_VO_4_, and a protease inhibitor mixture (Complete –EDTA tablets, Roche), and then centrifuged for 10 min at 4°C at 800×g to obtain a crude cytoplasmic supernatant fraction and a P1 pellet fraction containing nuclei and dense secondary lysosomes.

### Plasmids

The EGFP-HDQ74 construct expressing a truncated htt exon 1 fragment with 74Q fused to an EGFP reporter was described previously [44]. The EGFP-LC3 expression plasmid was a gift from T. Yoshimori, while the full-length 17Q-Htt construct was a gift from M. R. Hayden (described in [62]). The full-length 7Q-htt and ΔQ-htt expression constructs (Figure S5) were assembled from a genomic fragment containing mouse exon 1 with a portion of the flanking intron 1, a portion of the full-length mouse htt cDNA extending from exon 2 through exon 67 including a synthetic 3′splice acceptor site, and a poly(A) addition sequence from the bovine growth hormone gene. The mouse exon 1 fragments contained either wild-type sequences encoding the 7Q stretch, or sequences derived from our *Hdh^ΔQ^* targeting construct lacking the polyQ stretch. A 3×FLAG epitope tag was also inserted at the htt N-terminus between amino acids 1 and 2. A phosphoglycerol kinase (*pgk*) gene promoter was used to drive expression of the 7Q- and ΔQ-htt constructs in the transfected cells.

### Cell culture and transfection

SK-N-SH cells, wild-type Atg5 (*Atg5^+/+^*), and Atg5-deficient (*Atg5^−/−^*), HeLa cells, wild-type P5, and *Hdh^ΔQ/ΔQ^* P5 mouse embryonic fibroblasts (MEFs) were maintained in DMEM (D6546, Sigma) supplemented with 10% FBS, 100 U/ml penicillin/streptomycin and 2 mM L-glutamine (Sigma) in a 37°C, 5% CO_2_ humidified incubator. *Hdh^ex4/5^/Hdh^ex4/5^* knock-out (*Hdh^−/−^*) mouse ES cells were cultured on 0.1% gelatine coated tissue culture flasks in DMEM (D6546, Sigma) supplemented with 15% FBS, 1× L-Glutamine, 1× penicillin/streptomycin, 1× essential amino acids, 3.5 ml (per 500 ml media) 2-mercaptoethanol (Sigma) and 1000 U/ml ESGRO (ESG1107 with LIF, Chemicon/Millipore), and incubated in a 37°C, 5% CO_2_ humidified incubator. Cells were transfected with DNA constructs for 4 h using Lipofectamine 2000 (Invitrogen) according to the manufacturer's protocol, and either processed for western blotting analysis 24 h post-transfection by harvesting the cells and lysing the cell pellet on ice for 30 min in SDS-PAGE sample buffer (62.5 mM Tris-HCl pH 6.8, 2% SDS, 5% β-mercaptoethanol, 10% glycerol, 0.01% bromophenol blue) or fixed with 4% paraformaldehyde (Sigma) 48 h post-transfection and mounted with ProLong Gold antifade reagent containing 4′,6-diamidino-2-phenylindole (DAPI) (Invitrogen) for aggregation analysis.

### Western blotting

Samples (30 µg unless otherwise noted) were fractionated on SDS-PAGE, and then transferred electrophoretically onto 0.45 µm PVDF membranes (Invitrogen). Membranes were processed for western blotting using standard procedures. Antibody dilutions used were: rabbit polyclonal beclin 1 (H300; 1∶100, Santa Cruz Biotechnology; 1∶250), LC3 (1∶2,000 to 1∶5,000), guinea pig polyclonal p62/SQSTM1 (American Research Products; 1∶1,000), MW8 (1∶1,000), ubiquitin (DakoCytomation; 1∶1000), 1C2 (Chemicon/Millipore; 1∶5,000), anti-htt MAB2166 (Chemicon/Millipore; 1∶5000), β-actin (MP Biomedicals; 1∶50,000), and the following antibodies from Cell Signaling Technology used at 1∶1,000 dilution: rabbit monoclonal anti-Lamp1 (3243), rabbit anti-p70 S6 kinase (9202), rabbit anti-phospho-p70 S6 kinase (Thr^389^) (9205), rabbit anti-S6 ribosomal protein (2217), and rabbit anti-phospho-S6 ribosomal protein (Ser^235/236^) (2211). Blots were incubated 5 min in chemiluminescence reagent (SuperSignal West Dura, Pierce or an ECL detection kit, G.E. Healthcare) prior to film exposure. For densitometry, films in the linear exposure range were scanned on a flatbed scanner, and analyzed using the Image J program (Rasband, W.S., ImageJ, U.S. National Institutes of Health, Bethesda, MD, USA, http://rsb.info.nih.gov/ij/, 1997–2005). Levels of protein in each sample were normalized to actin, and the levels in the wild-type samples, with the exception of the mTOR/p-mTOR blots which were normalized to the band intensity of an abundant high-molecular weight protein visible on the blots after staining with Ponceau S.

### Statistical analyses

For behavioral tests, data was analyzed using the SigmaStat program (Systat Software). One-way ANOVA, two-way repeated measures ANOVA (with Bonferroni or Holm-Sidak post-hoc tests), and unpaired Student *t*-tests were used to analyze data. Significance was accepted at *P*<0.05. Mantel-Cox log rank tests on the Kaplan-Meier survival data were performed using SPSS 16.0.1 (SPSS Inc.). For quantification of htt aggregate number, lipofuscin deposit area, and levels of autophagy markers in subcellular fractions, Student *t*-tests were used. For analysis of LC3-II, S6K, and S6P levels in the in vitro cell culture experiments, a factorial ANOVA test using STATVIEW v4.53 (Abacus Concepts) was performed on the densitometric data, where the control condition was set to 100%. Error bars denote s.e.m. Pooled estimates for the changes in EGFP-HDQ74 aggregate formation resulting from perturbations assessed in multiple experiments, and the quantification of EGFP-LC3-positive vesicle numbers, were calculated as odds ratios with 95% confidence intervals. Odds ratios and *P* values were determined by unconditional logistical regression analysis, using the general log-linear analysis option of SPSS 9 software (SPSS Inc.), as previously described [42], [44]–[46],[61]. Experiments were performed in triplicate at least twice. ***, *P*< 0.001; **, *P*<0.01; *, *P*<0.05.

## Supporting Information

Figure S1 Reduced htt neuropil aggregates in the *Hdh^140Q/ΔQ^* cortex. (A) Confocal images of the parietal cortex from *Hdh^ΔQ/+^*, *Hdh^140Q/+^*, and *Hdh^140Q/ΔQ^* mice at 4 months, 6 months, 1 year, and 2 years of age (n = 4 of each genotype) immunostained with the MW8 antibody recognizing htt aggregates (red). Nuclei were stained with To-Pro-3 (blue). Enlarged images of the areas enclosed by the dashed white boxes are shown in the bottom panels. Scale bars = 25 µm (top panels), 10 µm (bottom three panels). (B) Total; T, nuclear; N, and neuropil; C, htt aggregate numbers from the *Hdh^140Q/+^* and *Hdh^140Q/ΔQ^* cortex (n = 4 of each genotype). The aggregate numbers represent counts/field (mean ± s.e.m.) from 8 images of the parietal cortex from each mouse. *P<0.05, **P<0.001.
(3.67 MB TIF)

Figure S2 ΔQ-htt expression enhances LC3 immunostaining in the 6 month old *Hdh^140Q/ΔQ^* striatum. Confocal images of LC3 (green) and htt aggregate (MW8, red) immunostaining in the striatum from 6 month old wild-type (+/+), *Hdh^ΔQ/+^*, *Hdh^140Q/+^*, and *Hdh^140Q/ΔQ^* mice (n = 4 of each genotype). Nuclei were stained with To-Pro-3 (blue). Enlarged images of the areas enclosed by dashed white boxes are shown in the bottom panels. Scale bars = 25 µm.
(2.71 MB TIF)

Figure S3 The lysosomal marker, Lamp1, is enriched in the *Hdh^ΔQ/+^* and *Hdh^140Q/ΔQ^* 800×g P1 fraction. (A) Striata dissected from wild-type (+/+), *Hdh^ΔQ/+^*, *Hdh^140Q/+^*, and *Hdh^140Q/ΔQ^* mice (n = 2 of each genotype) were homogenized and then centrifuged at 800×g, to generate a low-speed P1 fraction (see Methods). Aliquots of the P1 fraction were analyzed by western blotting using antibodies specific for lamp1 (marker for lysosomes and autolysosomes) and beclin 1 (an essential autophagy protein involved in autophagosome nucleation). Blots were then stripped and re-probed with a tubulin antibody (loading control). Both lamp1 and beclin 1 are enriched in the P1 fractions from the *Hdh^ΔQ/+^* and *Hdh^140Q/ΔQ^* striata, but are difficult to detect in the wild type and *Hdh^140Q/+^* fractions. (B) Striata dissected from wild-type, *Hdh^ΔQ/+^*, *Hdh^140Q/+^*, and *Hdh^140Q/ΔQ^* mice (n = 2 of each genotype) were homogenized and aliquots of the unfractionated extract were analyzed by western blotting using antibodies specific for LC3, beclin 1, and lamp1. Blots were then stripped and re-probed with a β-actin antibody (loading control).
(0.56 MB TIF)

Figure S4 Htt, calnexin, and LC3 localization in wild-type and *Hdh^ΔQ/ΔQ^* primary mouse embryonic fibroblasts. (A) Images of wild-type P5 (+/+) and *Hdh^ΔQ/ΔQ^* P5 primary mouse embryonic fibroblasts probed with an antibody specific for the ER marker calnexin (green), and an antibody recognizing both wild-type and ΔQ-htt (2166, red). Nuclei were stained with To-Pro-3 (blue). A merged image indicating overlap of the calnexin and htt immunoreactivity (orange to yellow color) is shown on the right. White arrowheads indicate increased nuclear htt immunoreactivity that correlates with a senescent cellular morphology. (B) Cells were probed with a mixture of calnexin (to visualize the ER; green) and FLAG antibodies (to visualize the N-terminal FLAG epitope tag on ΔQ-htt; red). The white arrowhead indicates increased nuclear ΔQ-htt immunoreactivity in an *Hdh^ΔQ/ΔQ^* senescent fibroblast. (C) Cells were probed with calnexin and LC3 antibodies to visualize ER (green), and autophagosomes (bright red punctate staining). Senescent cells exhibited increased perinuclear LC3 immunostaining. Scale bars = 10 µm.
(3.90 MB TIF)

Figure S5 Diagram of the 7Q-htt and ΔQ-htt expression constructs. A DNA fragment containing a synthetic 3′splice acceptor site, mouse htt cDNA sequence extending from exon 2 through exon 67, and a bovine growth hormone poly(A) addition site (located between the *Sph*I and *Kpn*I restriction sites) was cloned into the pGEM 5Zf plasmid vector (Promega). Inserted within the *Not*I restriction site located at the end of the synthetic splice acceptor site is a *Bam*HI to *Eco*RV fragment containing a phosphoglycerol kinase (pgk) gene promoter, an *Hdh* exon 1 genomic fragment containing either 7Q or ΔQ that was modified to contain a 3×FLAG epitope tag inserted at the htt N-terminus after the Methionine initiation codon, and a portion of the adjacent intron 1. Selected restriction sites are indicated, and the *Bam*HI restriction site within parentheses indicates that it was destroyed during cloning. N = *Not*I, A = *Apa*I, K or Kpn = *Kpn*I, X = *Xho*I, Xb = *Xba*I, Sp = *Sph*I, Nd = *Nde*I, B or Bam = *Bam*HI, Ns = *Nsi*I, R1 = *Eco*RI, X or Xmn = *Xmn*I. The orientation of transcription is indicated with an arrow.
(0.10 MB TIF)

Figure S6 ΔQ-htt expression in vitro increases the number of EGFP-LC3-positive vesicles. HeLa cells, transfected with EGFP-LC3 and either pCDNA3.1 (vector control), 7Q-htt or ΔQ-htt in a 1∶3 ratio for 4 h, were fixed at 24 h post-transfection. The proportion of EGFP-positive cells with >10 EGFP-LC3-positive vesicles was assessed and expressed as an odds ratio with 95% confidence limits. ΔQ-htt expression (***P<0.001), but not 7Q-htt expression (NS, *P* = 0.737), increased the proportion of cells with EGFP-LC3-positive vesicles compared to empty vector transfected cells. ΔQ-htt expression also increased the proportion of cells with EGFP-LC3 vesicles compared to 7Q-htt transfected cells (***P<0.001).
(0.03 MB TIF)

Figure S7 N-terminal htt fragments and htt aggregates are present in the *Hdh^140Q/ΔQ^* striatal pellet fraction. (A) Western blot analysis of the supernatants obtained following DNAse I digestion of a 16,100×g pellet fraction from *Hdh^140Q/ΔQ^* striatum (DNAse I), and the supernatants obtained following sequential extraction of the pellet (Pel) with buffers containing 0.1% Triton ×100 (Triton), CHAPS, and sodium deoxycholate (DOC). The blot in the top panel was probed with an antibody specific for the expanded polyQ stretch (1C2), while the bottom panel was probed with an antibody against p62/SQSTM1, a polyubiquitin-binding protein associated with htt aggregates [63], for comparison. A low level of soluble truncated htt fragments were recovered in the final pellet. (B) The pellet fractions from striata obtained from 2 year old wild-type (+/+), *Hdh^ΔQ/+^*, *Hdh^140Q/+^* (n = 1), and *Hdh^140Q/ΔQ^* (n = 2) mice were resuspended in SDS-PAGE sample buffer, fractionated by AGERA [64] on a 1% agarose gel, and analyzed by western blotting using an antibody recognizing htt aggregates (MW8, left panel), and an antibody recognizing ubiquitin (right panel). The position of monomeric protein, protein oligomers/aggregates, and the gel origin are indicated on the left. Note that htt aggregates are present in the *Hdh^140Q/ΔQ^* pellet fractions, but the amount of aggregated htt appears to be reduced compared to the levels in the *Hdh^140Q/+^* pellet fraction.
(1.22 MB TIF)
