# Supplementary figures and images for: Deletion of the Huntingtin Polyglutamine Stretch Enhances Neuronal Autophagy and Longevity in Mice
Source: PLoS Genet. 2010 Feb 5;6(2):e1000838. doi: 10.1371/journal.pgen.1000838 (PMC2816686; doi:10.1371/journal.pgen.1000838)

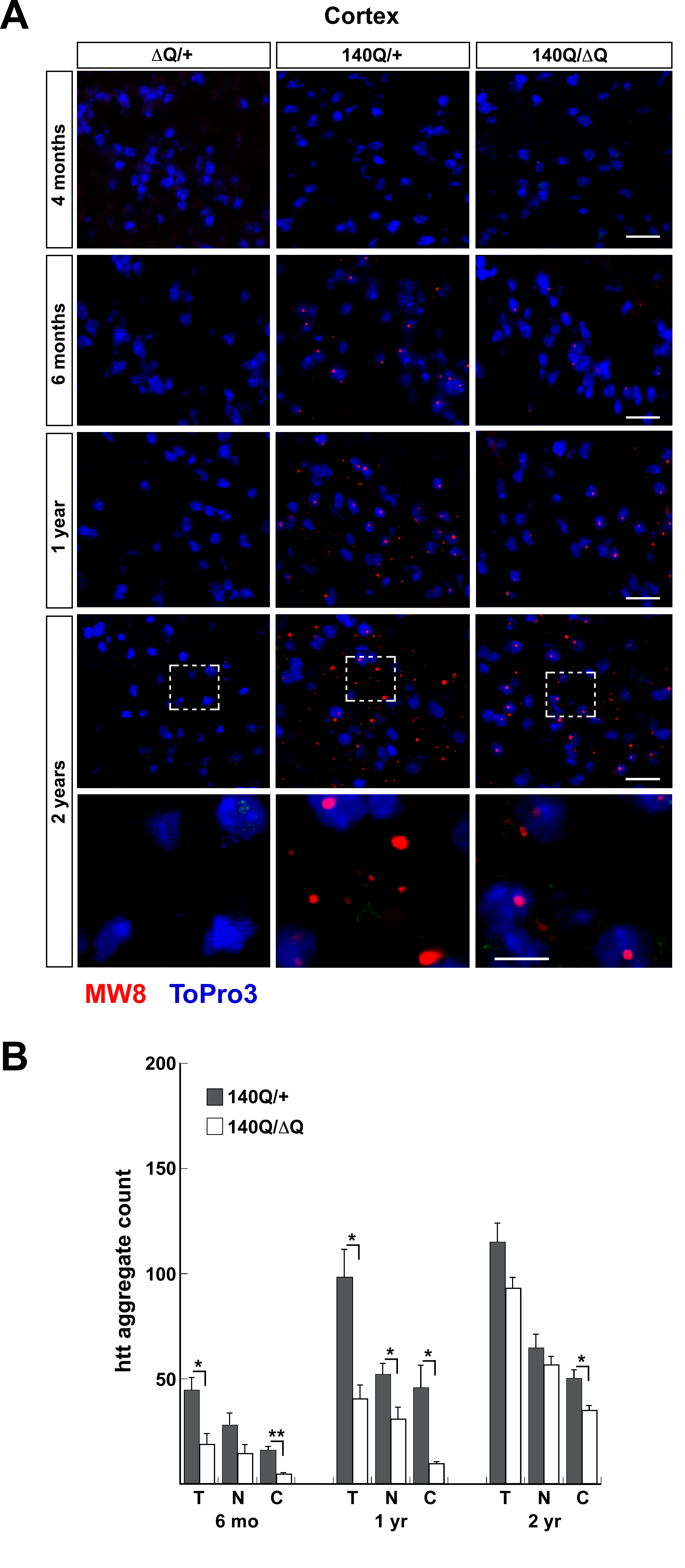

Supplement: Figure S1 — Reduced htt neuropil aggregates in the Hdh140Q/ΔQ cortex. (A) Confocal images of the parietal cortex from HdhΔQ/+, Hdh140Q/+, and Hdh140Q/ΔQ mice at 4 months, 6 months, 1 year, and 2 years of age (n = 4 of each genotype) immunostained with the MW8 antibody recognizing htt aggregates (red). Nuclei were stained with To-Pro-3 (blue). Enlarged images of the areas enclosed by the dashed white boxes are shown in the bottom panels. Scale bars = 25 µm (top panels), 10 µm (bottom three panels). (B) Total; T, nuclear; N, and neuropil; C, htt aggregate numbers from the Hdh140Q/+ and Hdh140Q/ΔQ cortex (n = 4 of each genotype). The aggregate numbers represent counts/field (mean ± s.e.m.) from 8 images of the parietal cortex from each mouse. *P<0.05, **P<0.001. (3.67 MB TIF) [file pgen.1000838.s001.tif]

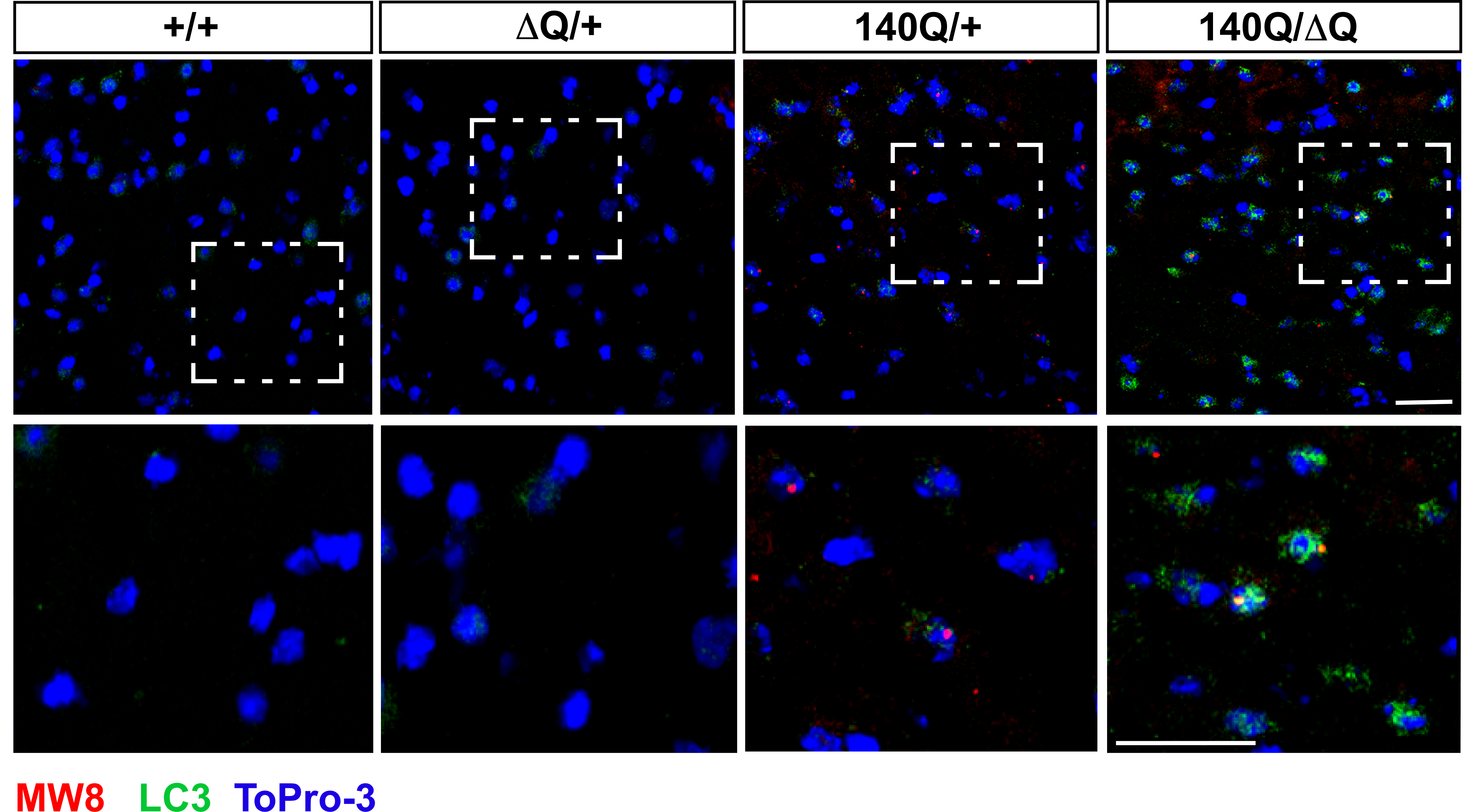

Supplement: Figure S2 — ΔQ-htt expression enhances LC3 immunostaining in the 6 month old Hdh140Q/ΔQ striatum. Confocal images of LC3 (green) and htt aggregate (MW8, red) immunostaining in the striatum from 6 month old wild-type (+/+), HdhΔQ/+, Hdh140Q/+, and Hdh140Q/ΔQ mice (n = 4 of each genotype). Nuclei were stained with To-Pro-3 (blue). Enlarged images of the areas enclosed by dashed white boxes are shown in the bottom panels. Scale bars = 25 µm. (2.71 MB TIF) [file pgen.1000838.s002.tif]

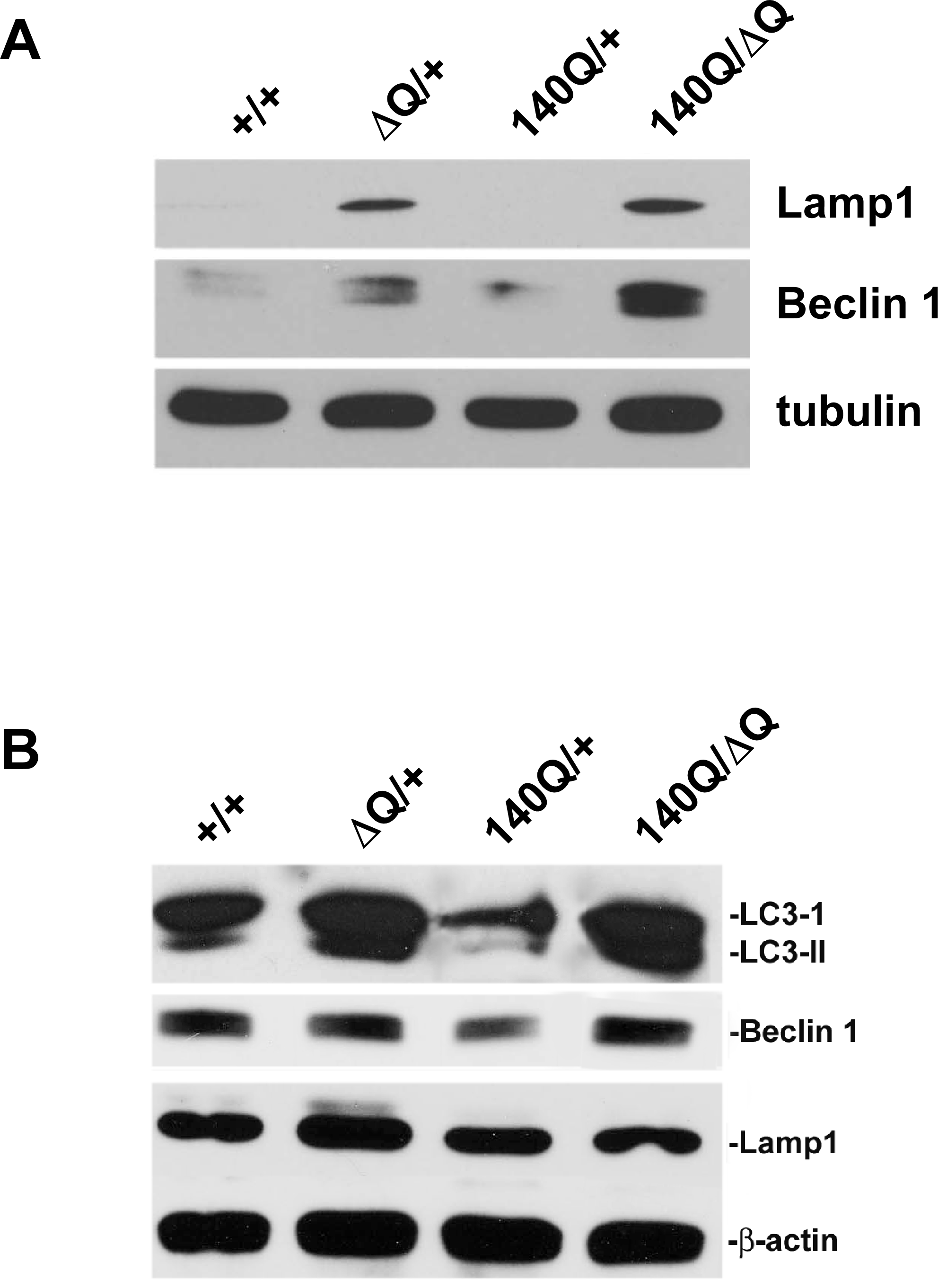

Supplement: Figure S3 — The lysosomal marker, Lamp1, is enriched in the HdhΔQ/+ and Hdh140Q/ΔQ 800×g P1 fraction. (A) Striata dissected from wild-type (+/+), HdhΔQ/+, Hdh140Q/+, and Hdh140Q/ΔQ mice (n = 2 of each genotype) were homogenized and then centrifuged at 800×g, to generate a low-speed P1 fraction (see Methods). Aliquots of the P1 fraction were analyzed by western blotting using antibodies specific for lamp1 (marker for lysosomes and autolysosomes) and beclin 1 (an essential autophagy protein involved in autophagosome nucleation). Blots were then stripped and re-probed with a tubulin antibody (loading control). Both lamp1 and beclin 1 are enriched in the P1 fractions from the HdhΔQ/+ and Hdh140Q/ΔQ striata, but are difficult to detect in the wild type and Hdh140Q/+ fractions. (B) Striata dissected from wild-type, HdhΔQ/+, Hdh140Q/+, and Hdh140Q/ΔQ mice (n = 2 of each genotype) were homogenized and aliquots of the unfractionated extract were analyzed by western blotting using antibodies specific for LC3, beclin 1, and lamp1. Blots were then stripped and re-probed with a β-actin antibody (loading control). (0.56 MB TIF) [file pgen.1000838.s003.tif]

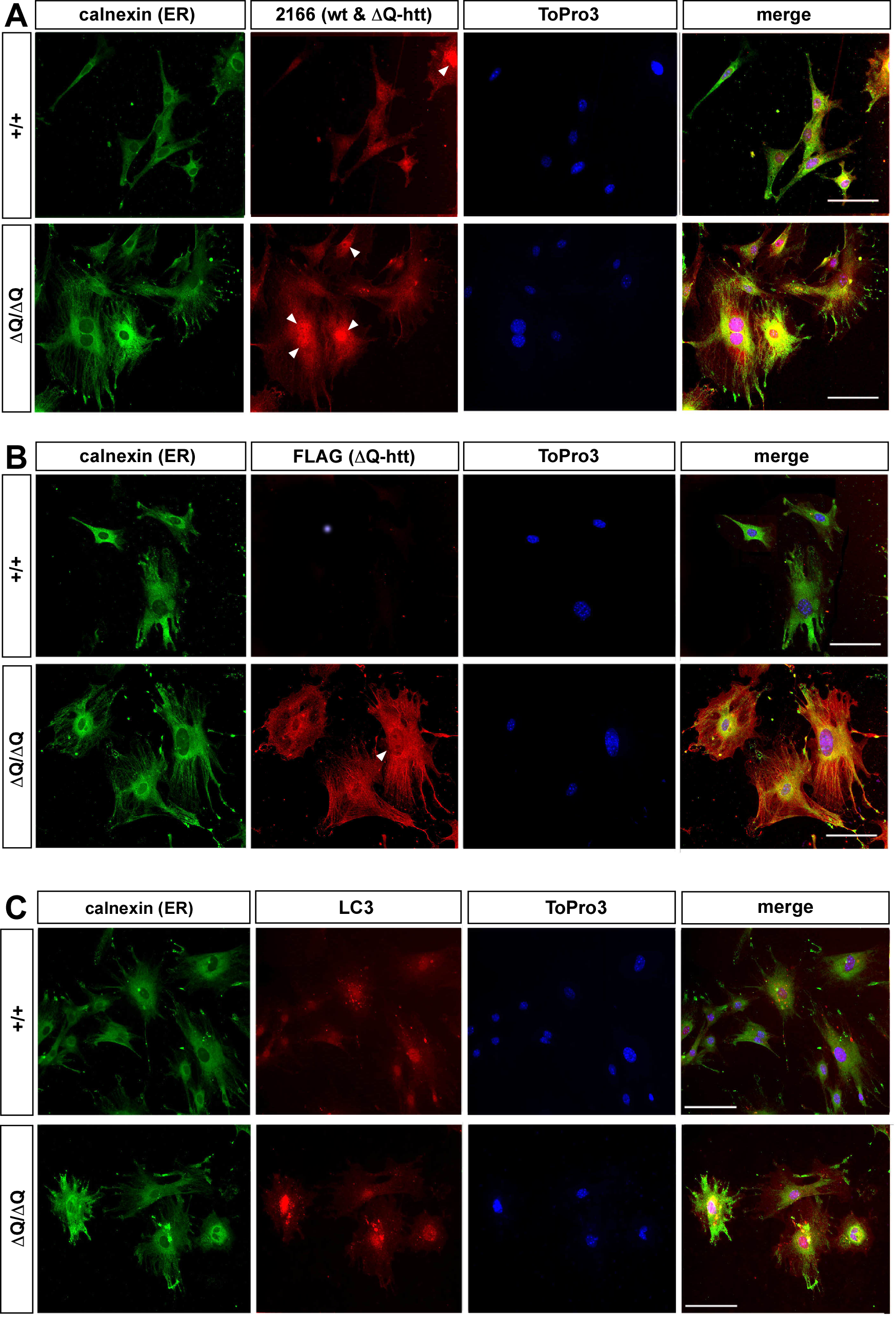

Supplement: Figure S4 — Htt, calnexin, and LC3 localization in wild-type and HdhΔQ/ΔQ primary mouse embryonic fibroblasts. (A) Images of wild-type P5 (+/+) and HdhΔQ/ΔQ P5 primary mouse embryonic fibroblasts probed with an antibody specific for the ER marker calnexin (green), and an antibody recognizing both wild-type and ΔQ-htt (2166, red). Nuclei were stained with To-Pro-3 (blue). A merged image indicating overlap of the calnexin and htt immunoreactivity (orange to yellow color) is shown on the right. White arrowheads indicate increased nuclear htt immunoreactivity that correlates with a senescent cellular morphology. (B) Cells were probed with a mixture of calnexin (to visualize the ER; green) and FLAG antibodies (to visualize the N-terminal FLAG epitope tag on ΔQ-htt; red). The white arrowhead indicates increased nuclear ΔQ-htt immunoreactivity in an HdhΔQ/ΔQ senescent fibroblast. (C) Cells were probed with calnexin and LC3 antibodies to visualize ER (green), and autophagosomes (bright red punctate staining). Senescent cells exhibited increased perinuclear LC3 immunostaining. Scale bars = 10 µm. (3.90 MB TIF) [file pgen.1000838.s004.tif]

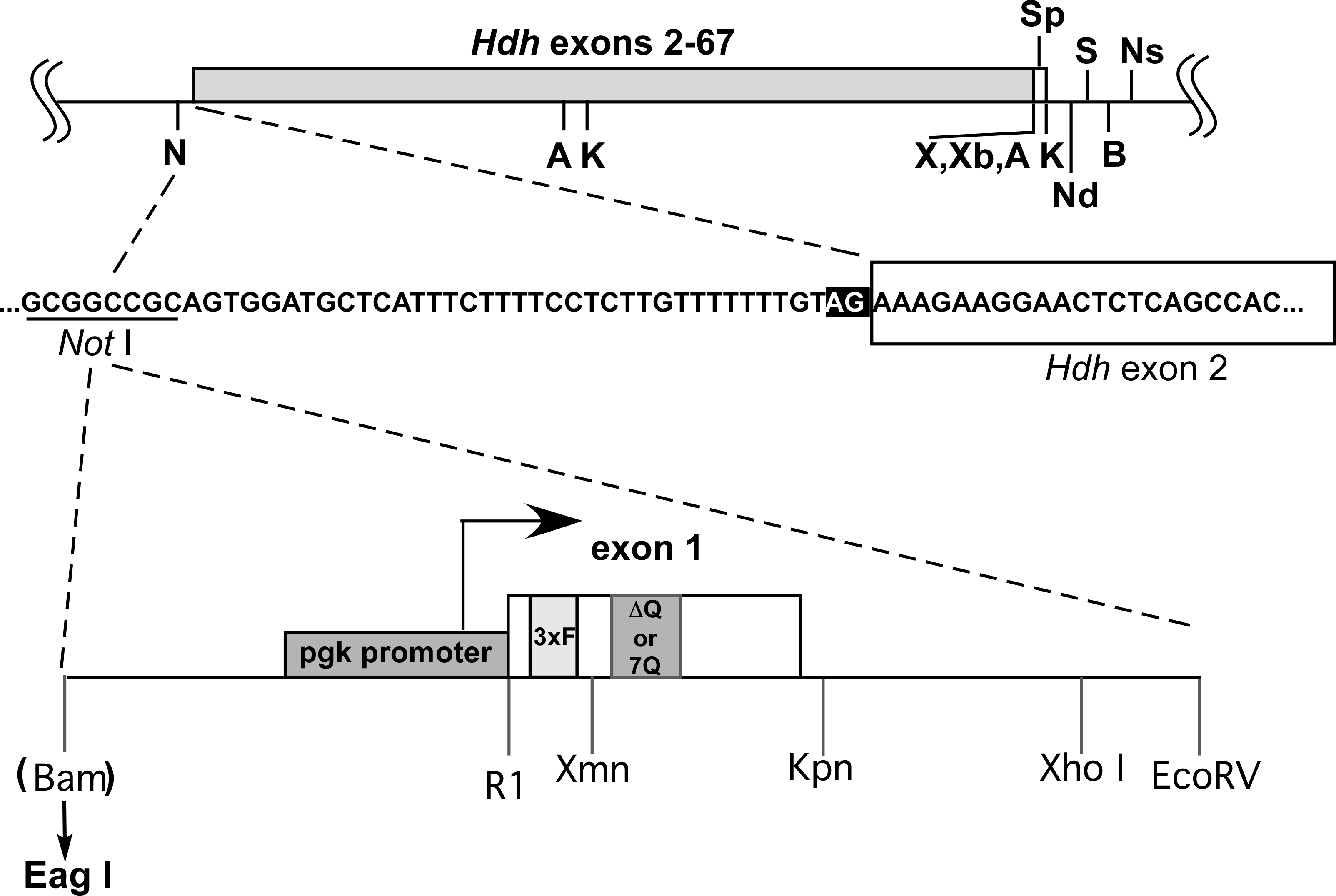

Supplement: Figure S5 — Diagram of the 7Q-htt and ΔQ-htt expression constructs. A DNA fragment containing a synthetic 3′splice acceptor site, mouse htt cDNA sequence extending from exon 2 through exon 67, and a bovine growth hormone poly(A) addition site (located between the SphI and KpnI restriction sites) was cloned into the pGEM 5Zf plasmid vector (Promega). Inserted within the NotI restriction site located at the end of the synthetic splice acceptor site is a BamHI to EcoRV fragment containing a phosphoglycerol kinase (pgk) gene promoter, an Hdh exon 1 genomic fragment containing either 7Q or ΔQ that was modified to contain a 3×FLAG epitope tag inserted at the htt N-terminus after the Methionine initiation codon, and a portion of the adjacent intron 1. Selected restriction sites are indicated, and the BamHI restriction site within parentheses indicates that it was destroyed during cloning. N = NotI, A = ApaI, K or Kpn = KpnI, X = XhoI, Xb = XbaI, Sp = SphI, Nd = NdeI, B or Bam = BamHI, Ns = NsiI, R1 = EcoRI, X or Xmn = XmnI. The orientation of transcription is indicated with an arrow. (0.10 MB TIF) [file pgen.1000838.s005.tif]

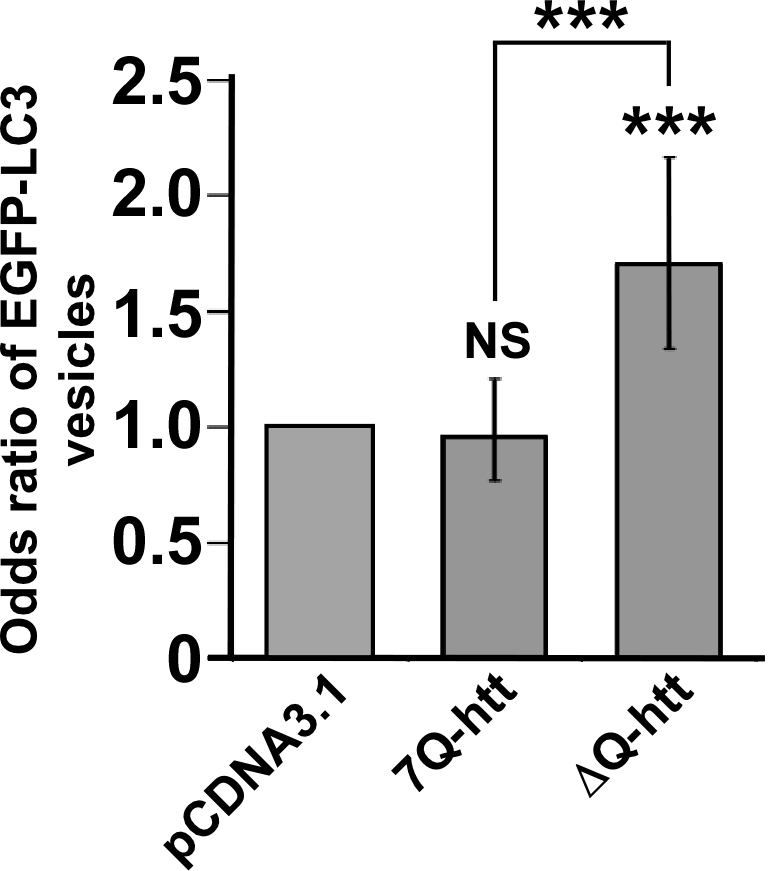

Supplement: Figure S6 — ΔQ-htt expression in vitro increases the number of EGFP-LC3-positive vesicles. HeLa cells, transfected with EGFP-LC3 and either pCDNA3.1 (vector control), 7Q-htt or ΔQ-htt in a 1∶3 ratio for 4 h, were fixed at 24 h post-transfection. The proportion of EGFP-positive cells with >10 EGFP-LC3-positive vesicles was assessed and expressed as an odds ratio with 95% confidence limits. ΔQ-htt expression (***P<0.001), but not 7Q-htt expression (NS, P = 0.737), increased the proportion of cells with EGFP-LC3-positive vesicles compared to empty vector transfected cells. ΔQ-htt expression also increased the proportion of cells with EGFP-LC3 vesicles compared to 7Q-htt transfected cells (***P<0.001). (0.03 MB TIF) [file pgen.1000838.s006.tif]

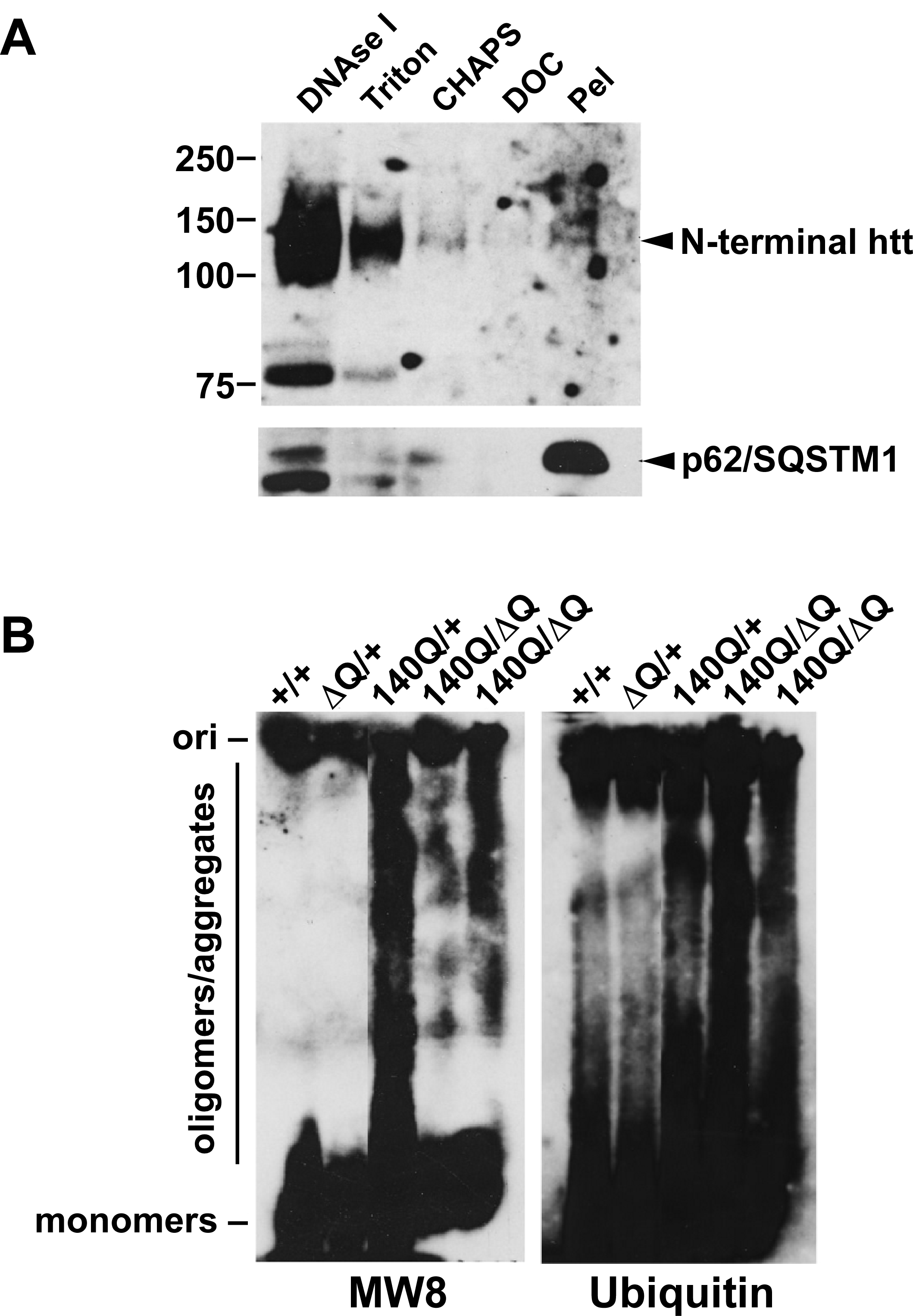

Supplement: Figure S7 — N-terminal htt fragments and htt aggregates are present in the Hdh140Q/ΔQ striatal pellet fraction. (A) Western blot analysis of the supernatants obtained following DNAse I digestion of a 16,100×g pellet fraction from Hdh140Q/ΔQ striatum (DNAse I), and the supernatants obtained following sequential extraction of the pellet (Pel) with buffers containing 0.1% Triton ×100 (Triton), CHAPS, and sodium deoxycholate (DOC). The blot in the top panel was probed with an antibody specific for the expanded polyQ stretch (1C2), while the bottom panel was probed with an antibody against p62/SQSTM1, a polyubiquitin-binding protein associated with htt aggregates [63], for comparison. A low level of soluble truncated htt fragments were recovered in the final pellet. (B) The pellet fractions from striata obtained from 2 year old wild-type (+/+), HdhΔQ/+, Hdh140Q/+ (n = 1), and Hdh140Q/ΔQ (n = 2) mice were resuspended in SDS-PAGE sample buffer, fractionated by AGERA [64] on a 1% agarose gel, and analyzed by western blotting using an antibody recognizing htt aggregates (MW8, left panel), and an antibody recognizing ubiquitin (right panel). The position of monomeric protein, protein oligomers/aggregates, and the gel origin are indicated on the left. Note that htt aggregates are present in the Hdh140Q/ΔQ pellet fractions, but the amount of aggregated htt appears to be reduced compared to the levels in the Hdh140Q/+ pellet fraction. (1.22 MB TIF) [file pgen.1000838.s007.tif]
